# Free quantum computing

**DOI:** 10.1073/pnas.2510881123

**Published:** 2026-02-17

**Authors:** Jacques Carette, Chris Heunen, Robin Kaarsgaard, Neil J. Ross, Amr Sabry

**Affiliations:** ^a^Department of Computing and Software, McMaster University, Hamilton, ON L8S 4K1, Canada; ^b^School of Informatics, University of Edinburgh, Edinburgh EH9 1DF, United Kingdom; ^c^Department of Mathematics and Computer Science, Centre for Quantum Mathematics, Centre for Formal Methods and Future Computing, University of Southern Denmark, Odense 5230, Denmark; ^d^Department of Mathematics and Statistics, Dalhousie University, Halifax, NS B3H 4R2, Canada; ^e^Department of Computer Science, Indiana University, Bloomington, IN 47408

**Keywords:** reversible computing, axiomatization, free model, category theory

## Abstract

Quantum computing improves substantially on known classical algorithms for various important problems, but the nature of the relationship between quantum and classical computing is not yet fully understood. This relationship can be clarified by free models, that add to classical computing just enough physical principles to represent quantum computing and no more. Here, we develop an axiomatization of quantum computing that replaces the standard continuous postulates with a small number of discrete equations, as well as a free model that replaces the standard linear-algebraic model with a category-theoretical one. The axioms and model are based on reversible classical computing, isolate quantum advantage in the ability to take certain well-behaved square roots, and link to various quantum computing hardware platforms. This approach allows combinatorial optimization, including brute force computer search, to optimize quantum computations. The free model may be interpreted as a programming language for quantum computers, that has the same expressivity and computational universality as the standard model, but additionally allows automated verification and reasoning.

## Significance

Quantum computing holds great promise, but its foundations and the source of its advantages remain conceptually obscure. We develop a framework that contains no extraneous mathematical assumptions and clearly separates what is truly quantum from what is just classical computing in disguise. Instead of relying on the infinite precision of continuous complex numbers, this symbolic approach uses a small finite number of discrete building blocks that reflect physical implementations. Unlike traditional models, this model supports purely symbolic combinatorial reasoning, enabling the use of powerful classical computer science techniques. This framework is just as effective as traditional ones, and offers a rigorous, simpler foundation to understand and engineer quantum computation.

Quantum computing improves substantially on known classical algorithms for certain problems (1) including prime factorization (2), boson sampling (3), and Hamiltonian simulation (4). The nature of the relationship between classical and quantum computing is not yet fully understood. The literature only identifies several notions that do not explain the difference on their own (5), including superposition (6), entanglement (7), nonlocality (8), contextuality (9), and interference (10). The advantage of quantum computing over classical computing is often considered quantitatively: just how much advantage does a specific quantum algorithm have over classical ones (11)? Here, we consider it qualitatively: does a model of computation allow algorithms that have advantage over classical ones, or does it not?

The question is difficult partly because quantum computing is often implicitly confined to a standard model, that uses complex linear maps (12). For a meaningful comparison, both classical and quantum computing must be situated within a larger landscape of models. We use as models bipermutative categories, the most permissive notion of model of computation possible. Within this framework, we identify a unified free model of quantum computing that can express all quantum algorithms with advantage over classical ones.

**Fig. 1.**
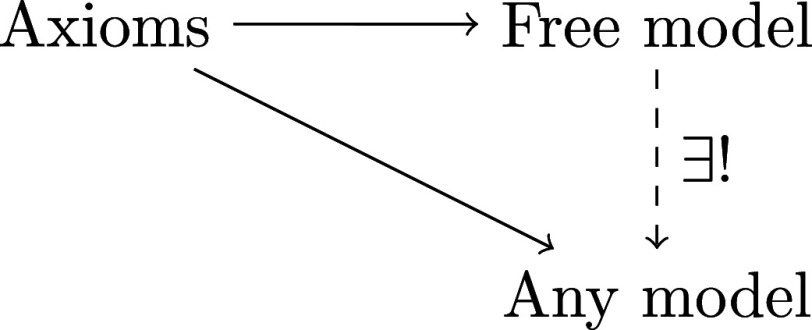
The defining property of a free model: it realizes our axioms of quantum computation, and there is a unique interpretation of the free model in any other model satisfying our axioms. In this sense, the free model contains exactly what is needed to form a model, and nothing more.

To explain the free property of a model by way of example, consider combinatorics. The natural numbers can be completed with negatives in multiple ways: N embeds into Z while preserving addition as n↦n, but also as n↦−n or n↦2n. The fact that these are completions with negatives means that an additive inverse for the number 1 is adjoined, and entails that all these embeddings are in fact the same up to an unimportant global scalar. The fact that it is the free completion means that any other completion has to encompass it. The real numbers also contain N and allow subtraction, but additionally have many other properties that are superfluous for counting. Any addition-preserving embedding N→R factors uniquely through the free model Z (of natural numbers with negatives). In this sense, the integers are the smallest model, devoid of extraneous assumptions or constraints, of natural numbers with negatives. This is exactly what being a free model captures. See Fig. 1. Generally, free models are unique: they are completely determined by their defining properties (such as having negatives) up to a unique isomorphism (such as an unimportant global scalar).

This article explains the relationship between classical and quantum computing in a similar way. Like adjoining −1 to the natural numbers, we adjoin a small number of generators with physical significance to reversible classical computing and show that reversible quantum computing arises as the free completion. Like the real numbers, any other model of reversible quantum computing, including the standard model of complex unitary matrices, must factor uniquely through this free one. Conversely, we can canonically identify classical computing within any model of quantum computing by the latter’s (bipermutative) structure. The free model has the same computational universality as the standard model: it can express any quantum algorithm to any desired accuracy. But it has additional virtues.

The main advantage of this free model over the standard model is that it enables automated reasoning about quantum algorithms. For example, deciding whether two quantum circuits represent the same quantum computation is an intractable problem (13), among other reasons because there are continuously many degrees of freedom. But because the free model is entirely discrete and concerns only finitely many equations, the full power of combinatorial optimizations and computer science methods of equational rewriting can be brought to bear (14). As a simple example, we will prove that the Hadamard gate is an involution; this fact is not deep, but the point is that this equality can be derived automatically, as can all others.

A second advantage of the free model is foundational: it does not conflate physical concepts. We thus isolate the difference between quantum and classical computation in the ability to take well-behaved square roots, or in other words, the ability to stop some classical computations half-way. The free model is explained from operational first principles, unlike the foundationally unsatisfactory reliance on complex numbers (15). Similarly, it does not need or allow intermediate unphysical matrices that have to be made unitary again at a later stage, circumventing the inefficiency of circuit synthesis that plagues other approaches (16).

A third advantage of the free model is practical: it does not resort to unempirical numbers that cannot experimentally be determined with arbitrary precision in finite time, but accounts for accuracy precisely. We will not discuss quantitative results in individual models of computation, like the density theorems (17, 18) that show how efficiently a unitary matrix can be approximated by quantum circuits. Nevertheless, as an example we will analyze the relationship between accuracy in the free model and efficiency of its implementation of the quantum Fourier transform.

A substantial literature has explored structural and equational approaches to quantum computation, most notably categorical quantum mechanics (19, 20) and diagrammatic formalisms such as the ZX-calculus (21). These frameworks replace matrices by generators and rewrite rules, enabling symbolic manipulation and circuit optimization. Our aim is related but conceptually distinct. Rather than giving sound and often complete presentations of specific models—typically Hilbert spaces or fragments thereof—we construct the free model generated by a set of axioms with physical connotations. The result is not a calculus for reasoning within a fixed quantum model, but a universal quantum programming language for that axiomatization.

## Auxiliary Qubits

**Fig. 2.**
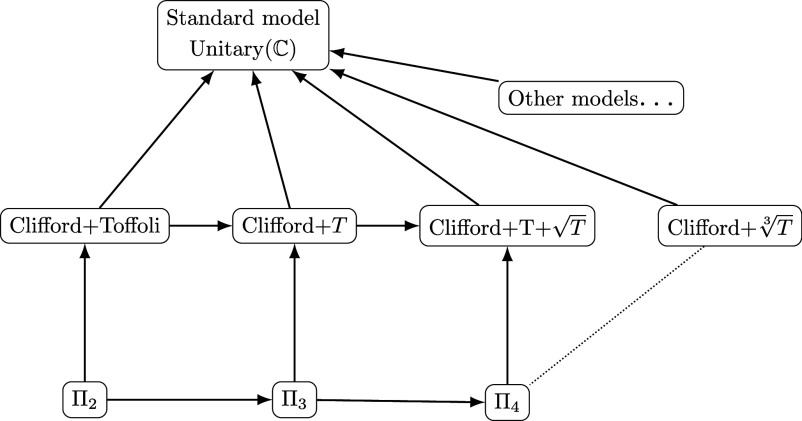
The landscape of models of quantum computing and their relations. Solid arrows indicate inclusion of one model into another. Incomparable models are connected with dotted lines. For each k, $\Pi_{k}$ is the free model for the axioms of bipermutative categories augmented with axioms [**1**]–[**3**]. The standard model of unitaries over the complex numbers also satisfies these axioms. For each k, $\Pi_{k}$ embeds in any model that also satisfies the same axioms. For another example, the model of Clifford+$\sqrt[{3}]{T}$ circuits is also computationally universal (24) but incomparable to any $\Pi_{k}$.

The quantum computing literature focuses mostly on universality of finite gate sets, rather than explicit comparison to classical computation (22). Here, our focus is on models of quantum computation that reuse the infrastructure of classical computation and are reached by a finite set of axioms. We fix one set of axioms, containing square roots and a precision parameter k, and investigate its free model. We show that the precision level can be increased by n at the cost of n auxiliary qubits. For k=2 the free model encompasses Clifford+Toffoli quantum circuits; k=3 encompasses Clifford+T quantum circuits; k=4 encompasses Clifford+T+$\sqrt{T}$ quantum circuits; and higher precision encompasses Clifford cyclotomic quantum circuits (23). Other axioms, for example using cube roots, give other free models. See Fig. 2. In general it makes no sense to compare models for different axioms, but we conjecture that as k tends to infinity, the free model of the axioms using square roots coincides with that using cube roots or roots for any other radix. Our axioms distinguish themselves by having physical connections beyond the mathematical and computational advantages discussed above.

In addition to increased precision, auxiliary qubits also provide the opportunity to extend our free model of reversible quantum computing with quantum measurement. A second free construction allows mid-computation measurements to control the flow of the quantum computation (25). We will show that the ability to measure out auxiliary qubits strengthens the completeness of our free model: two irreversible quantum computations are equal if and only if they follow from our axioms, without any need for auxiliary qubits.

## Axioms

The standard postulates of reversible quantum computing may be replaced by the following; we will discuss (irreversible) quantum measurements later. The axioms of reversible quantum computing, with precision level k, extend reversible classical computing with two generators $\zeta_{k}$ and V and three relations

[1] $$\begin{array}{cc} V^{2} & =X \end{array}$$

[2] $$\begin{array}{cc} V\;S\;V & =S\;V\;S \end{array}$$

[3] $$\begin{array}{cc} \zeta_{k}^{2^{k}} & =1, \end{array}$$

where X is a coherent quantum version of the classical NOT gate, and S is defined in terms of $\zeta_{k}$, as explained in more detail below (26). Classical reversible computing corresponds to the free bipermutative category with no further assumptions, which embeds into any other bipermutative category.

These axioms isolate (well-behaved) square roots as a singular source of any difference between quantum computing and classical computing. We will also consider a weaker form of equivalence $\approx_{k}$ which identifies computations when they are equal in the presence of auxiliary qubits. This weak equivalence becomes irrelevant when including (irreversible) quantum measurement and state preparation.

## Interpretation

Before we go into detail, observe the advantages of this axiomatization. It consists of a finite number of equations, without continuous variables or logical quantifiers. Therefore they are eminently amenable to automation, and can benefit from combinatorial optimization, including brute force computer search (27).

Moreover, all three equations have direct realizations in important physical models of computation, along with links to conceptual principles, notably superposition and interference.

Axiom [**1**] can be read as saying that the X gate has a square root. This is enacted in trapped-ion quantum computing by pulsing the laser for half the duration or intensity needed to enact the X gate (28). Similarly, $\sqrt{X}$ is a native gate in superconducting quantum computers (1). The X gate having a square root induces superposition, but here, the ability to halt some computations halfway is primary, and the existence of some superpositions is merely derived. Constructing all superpositions is impossible in a model that extends classical reversible computing with only finitely many axioms.

**Fig. 3.**
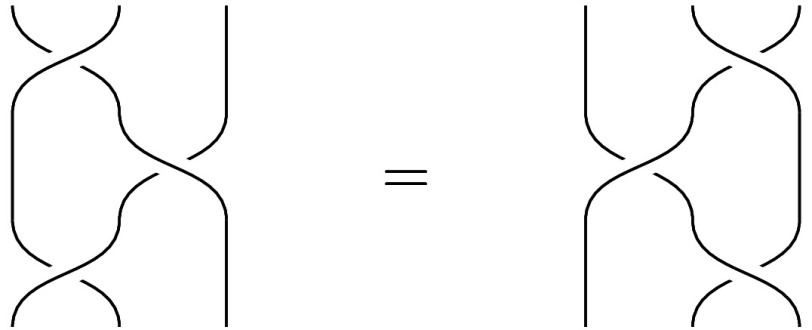
An illustration of Axiom [**2**], interpreting V as swapping the leftmost two wires, and S as swapping the rightmost two wires.

Axiom [**2**] is the defining equation for the 3-strand braid group, linking this axiomatization to anyonic quantum computing (29). See Fig. 3.

Axiom [**3**] concerns constructivity. General complex numbers in principle take forever to determine with infinite precision. The free model instead uses numbers that one can empirically construct or measure exactly. Positing a nontrivial phase, in combination with the superposition afforded by V, induces interference. We choose a primitive $2^{k}$th root of unity $\zeta_{k}=e^{2\pi i/2^{k}}$ as our primitive nontrivial phase. The scalars in the free model (as defined in “Bipermutative categories: formalizing the main results” below) are therefore the cyclic group of order $2^{k}$, consisting of powers of $\zeta_{k}$. One can clearly approximate any complex number on the unit circle with such constructive scalars by choosing k sufficiently large, additionally making these scalars useful in parametrized rotation gates, photonic quantum computing, and variational quantum algorithms. Notice that the free model has no need for addition of scalars, but the standard model of unitary matrices uses many more scalars.

## Reversible Classical Computing

To understand the axiomatization and explain what we mean by a *model* in more detail, we first discuss reversible classical computing. In this paradigm, computations are deterministic when run either forward or backward (30). It relates to physics, as experiments have verified that irreversibly changing information stored in a computer dissipates heat (31), in accordance with Landauer’s principle (32, 33).

The standard model of classical reversible computing is given by circuits of reversible Boolean gates. For example, the gate CCX, also known as the Toffoli gate, which transforms three input bits $\left(x,y,z\right)\in {\left\{0,1\right\}}^{3}$ into (x,y,xy+zmod2), is universal in the sense that any Boolean function can be implemented by a circuit of CCX gates.

A general model for classical reversible computing is given by the notion of a bipermutative category, which has objects and processes that can be combined using combinators ⊕ and ⊗ (34). This is the bare minimum that any model of computation needs to represent: the ability to compose instructions sequentially (using ○), the ability to consider data in parallel (using ⊗), and the ability to use one such concurrent piece of data to influence another (using ⊕). In the standard model, the unit object 1 for ⊗ is analogous to a singleton set, and 2=1⊕1 models the bit. The swap process X:1⊕1→1⊕1 models a NOT gate, and CCX is typical of modeling controlled operations as id⊕X:2⊕2⊕2⊕2=2⊗2⊗2→2⊗2⊗2.

The free model of classical reversible computing consists of permutations of finite sets, or equivalently, permutation matrices. Because every permutation of a finite set is a composition of transpositions, the NOT gate X generates all of these. The free bipermutative category is thus identified with the free model of classical reversible computing: the X and CCX gates are already modeled by the axioms of bipermutative categories.

## Reversible Quantum Computing

Similar combinators ⊕ and ⊗ extend quantum computing from a single qubit to multiple controlled qubits. We can now understand Axioms [**1**]–[**3**] in detail. Start with the free bipermutative category, i.e. the free model of classical reversible computing, and choose an integer k≥2. Add generators $\zeta_{k}:1\to 1$ and V:2→2, and consider the free bipermutative category satisfying Axioms [**1**]–[**3**], where $S=\text{id}⊕\zeta_{k}^{2^{k-2}}$. *Theorem* 1 below shows that this free model exists. Notice that if we only used ⊗, adding $\zeta_{k}$ merely yields physically irrelevant global phases; it is the presence of ⊕ that yields relative phases with nontrivial computational consequences. The combinator ⊕ gives the ability to represent qudits for all dimensions rather than only qubits, which harmonizes with the need for quantum algorithms such as Shor’s prime factoring to allocate storage for values to the nearest power of 2, some of which will not be used in the actual computation.

The standard model of unitary matrices also satisfies Axioms [**1**]–[**3**] with

$$\zeta_{k}=e^{2\pi i/2^{k}}\text{,}\;V=\frac{1}{2}\left(\begin{array}{cc} 1+i & 1-i\\ 1-i & 1+i \end{array}\right)\text{,}\;S=\left(\begin{array}{cc} 1 & 0\\ 0 & i \end{array}\right).$$

Hence there is an interpretation of the free model in the standard matrix model (Fig. 1). *Theorem* 2 below shows that this interpretation is in fact an isomorphism when k=2 to a subset of the standard matrix model. Free models are unique up to unique isomorphism: given two free models, setting one to be the “Free model” and instantiating “Any model” in Fig. 1 to be the other and vice versa gives two maps that are each other’s inverse. We conclude that the free model (which has k=2) is formed by unitary matrices with entries from the smallest ring containing $\zeta_{2}$ and $\frac{1}{2}$. Vice versa, for each k, we may think of the free model as a programming language with which to construct reversible quantum programs.

The standard matrix interpretation answers two computer science questions (35) about this quantum programming language. First, the word problem is decidable: given two expressions in the programming language, there is an algorithm that can decide whether they compute the same function, by comparing the matrices they induce. Second, normalization by evaluation (36) is possible: one can optimize a quantum program by synthesizing a normal form from the matrix it induces. This is akin to quantum circuit optimization.

Finally, by foregoing matrices and working with the free model no reasoning power is lost. It follows from *Theorem* 2 below that two quantum programs denote the same matrix if and only if they are provably equal under substitutions using Axioms [**1**]–[**3**]. It thus becomes possible to automate verification of quantum programs, and to use automated proof assistants (37) to construct, transform, and reason about quantum computations.

## Precision and Conditional Circuits with Auxiliary Qubits

*Theorem* 3 establishes that for every k≥2, the free model is topologically dense in the standard model of unitary matrices over the complex numbers. In other words, any target unitary can be approximated to arbitrary accuracy by a program in the free model, justifying its status as a model of (reversible) quantum computation.

*Theorem* 4 further shows that the precision parameter k does not affect expressivity once a clean auxiliary qubit is available: $\Pi_{k+1}$ can be simulated exactly by $\Pi_{k}$ using a single auxiliary qubit. Thus k only influences the size and convenience of terms, not which unitaries are definable. In particular, k=2 already suffices for universal approximation. Larger k can produce much shorter $\Pi_{k}$ terms for the same target unitary, and can make it easier to reason about programs and prove algebraic properties within the syntax, since fewer identities must be expressed through long compiled sequences. Indeed, certain highly structured unitaries benefit dramatically from having more primitive phases. A notable example is the quantum Fourier transform (QFT), whose phase angles are exact powers of 1/2, making it an ideal case study for the impact of k on synthesis cost.

Each n-qubit QFT contains $O(n^{2})$ controlled-phase gates with angles $2\pi /2^{d}$ for 2≤d≤n (12). Because $\Pi_{k}$ includes all phases with denominator $2^{k}$ as primitives, only smaller-angle rotations require approximation, at a cost of $O({\text{log}}_{2}\left(1/\varepsilon \right))$ per rotation, where ε is the accuracy of the approximation (18, 38). Increasing k reduces the number of rotations to approximate until k reaches n, after which all rotations are native and the size plateaus at $O(n^{2})$. This sharp cutoff is a consequence of the QFT’s structured angles; for generic unitaries, which lack such alignment, increasing k can only improve constant factors and does not remove the O(log(1/ε)) synthesis overhead.

Beyond reducing the cost of phase synthesis, the $\Pi_{k}$ formalism also yields smaller controlled circuits. Recent compilation techniques show how to decompose multicontrolled operations linearly without helper qubits, by exploiting higher-order roots and reusing idle qubits as temporary workspace (39). This means that increasing k not only shortens the phase-synthesis portion of a program, but also reduces the overhead of conditionals and controlled subroutines, further amplifying the practical benefits of higher precision.

## Measurement

Finally, we discuss a free replacement of the standard postulate about measurement, by incorporating (mid-computation) quantum measurement, initialization of auxiliary qubits, and classical control into the above account of reversible quantum computing. Measurement necessitates working with mixed rather than pure states. This means that the objects of the free model can no longer be natural numbers modeling dimensions. Instead, they will be lists of natural numbers, modeling dimensions branched on measurement outcomes, enabling classical control.

An irreversible quantum computation from m to n qubits can be simulated perfectly by a reversible quantum computation from m+n to m+n qubits using quantum information effects (25). This is another free construction that introduces the ability to hide information in a way that respects both sequential composition (○) and parallel composition (⊗) according to the einselection interpretation of decoherence (40). It may be thought of as a further programming language, in which auxiliary qubits, classical control, and measurements can be used at will anywhere in the quantum program, that gets compiled into a quantum program with a single measurement at the end. *Theorem* 5 below states that two such programs including measurement denote the same quantum channel if and only if they are provably equal under substitutions using a finite set of equations—see *SI Appendix* for details. The physical irrelevance of global phase arises in this construction from the uniformity of information hiding, squashing all global phases into a single point.

Similar to how mixed states of n qubits correspond to quantum channels $C\to {\left(C^{2}\right)}^{⊗n}$, states of some object A in the free model correspond to morphisms 1→A. An input state ρ:1→A evolves along an operation f:A→B by composition to give output state f○ρ:1→B. Probabilities are thus a derived concept in the free model, with mixed states given by the equivalence class of their purifications.

## Bipermutative Categories: Formalizing the Main Results

Let us now sketch the technical development, leaving full detail to *SI Appendix*. A category consists of objects and morphisms between those objects, such that morphisms f:A→B and g:B→C can be composed to g○f:A→C (typically shortened to gf) in an associative way, and there are identity morphisms id:A→A on each object (41). Categories can fully characterize quantum theory without referring to the standard model (42). A strict symmetric monoidal category additionally has objects A⊗B for any two objects A and B, and morphisms f⊗g:A⊗B→C⊗D for any morphisms f:A→C and g:B→D, a unit object 1 with 1⊗A=A, and a swap morphism σ:A⊗B→B⊗A, such that (id⊗g)○(f⊗id)=(f⊗id)○(id⊗g) and σ○σ=id (20). Morphisms s:1→1 are called scalars, any morphism f:A→B can be scaled by a scalar s to form s·f:A→B, and this scalar multiplication commutes with all other operations (20). A bipermutative category is a category that is strict symmetric monoidal in two ways, (⊗,1) and (⊕,0), such that (A⊕B)⊗C=(A⊗C)⊕(A⊗B) and three natural equations hold (43). A category is free with some property when it satisfies the condition of Fig. 1 (41).

Theorem 1 *There exists a free bipermutative category* $\Pi_{k}$ *with generators* $\zeta_{k}:1\to 1$ *and* V:1⊕1→1⊕1 *satisfying Axioms [**1**]–[**3**]*.

Recall that, like any free model, $\Pi_{k}$ is unique up to unique isomorphism. For example, $\Pi_{0}$ is the category whose objects are natural numbers, whose morphisms are permutations of {1,…,n}, and which only has one trivial scalar. Another example of a bipermutative category is **Unitary**(*R*), where objects are natural numbers, morphisms are unitary matrices over the involutive ring R, and scalars are elements of R. For example, R could be the ring of dyadic rational numbers $D=Z\left[\frac{1}{2}\right]=\left\{2^{-n}z|n\in N,z\in Z\right\}$, with a primitive $2^{k}$th root of unity $\zeta_{k}$ adjoined.

Theorem 2 *There is an isomorphism* $\Pi_{2}≃Unitary\left(D\left[\zeta_{2}\right]\right)$ *of bipermutative categories*.

***Proof sketch:*** Freeness of $\Pi_{2}$ gives a functor from $\Pi_{2}$ to $Unitary(D\left[\zeta_{2}\right])$ that is bijective on objects. That it is bijective on morphisms follows because $\Pi_{2}$ satisfies the equations generating $Unitary(D\left[\zeta_{2}\right])$ (26).

Just as a quantum circuit is merely a formal diagram, morphisms in $\Pi_{k}$ are the source code of a quantum program. To physically perform the computation, a meaning has to be assigned to it, just like how quantum circuits are interpreted as unitary matrices. This assignment of meaning preserves the structure of computations.

Theorem 3 *There is an inclusion* [[−]] *from* $\Pi_{k}$ *into* Unitary(C) *for any precision level* k, *and for* k≥2 *its image is dense*.

***Proof sketch:*** Already at precision k=2, the category $\Pi_{2}$ contains the CCX gate as well as all Clifford gates (up to a global phase). This suffices to approximate arbitrary complex unitary matrices without auxiliary qubits (44).

Similarly, a translation between bipermutative categories is a function that preserves ○ and ⊕.

Theorem 4 *There is a translation* Φ *from* $\Pi_{k+1}$ *to* $\Pi_{k}$ *for* k≥2 *such that* f *and* g *in* $\Pi_{k+1}$ *satisfy* [[f]]=[[g]] *if and only if* [[Φ(f)]]=[[Φ(g)]] *in* $\Pi_{k}$.

***Proof sketch:*** This construction generalizes (22) by emulating a square root of a given scalar using one auxiliary qubit, and noticing that $\Pi_{k+1}$ satisfies this.

*SI Appendix* details a free construction that adds initialization morphisms, decoherence morphisms, and classical control to a bipermutative category $\Pi_{k}$ to turn it into a strict monoidal category $\text{Split}(\mathrm{LR}\;\left(\Pi_{k}\right))$ (25), and an equivalence $\approx_{k}$ that identifies computations when they are equal in the presence of auxiliary qubits.

Theorem 5 *There is an inclusion* [[−]] *from* $\text{Split}(\text{LR}\left(\Pi_{k}\right))$ to the category of completely positive linear maps between finite-dimensional C*-algebras for any precision level k, *and* [[f]]=[[g]] *if and only if* f=g *in* $\text{Split}(\mathrm{LR}\;\left(\Pi_{k}\right))$. *Computations* f *and* g *in* $\Pi_{k}$ *are equal in* $\mathrm{Split}\;(\mathrm{LR}\;\left(\Pi_{k}\right))$ *if and only if* $f\approx_{k}g$ *up to a global phase*.

***Proof sketch:*** The category of quantum channels has a known presentation (45), and it suffices to verify that $\text{Split}(\mathrm{LR}\;\left(\Pi_{k}\right))$ satisfies its equations.

## Reasoning

Reasoning in $\Pi_{k}$ is exact, symbolic, and complete. Even for a seemingly trivial identity such as HH=id, standard approaches face difficulties. One option is to work with matrices whose entries involve algebraic numbers, making exact proofs extremely delicate and number-theoretic in nature. While there are decision procedures for these, their complexity is unwieldy. The fundamental issue is that the theory of algebraic numbers cannot be finitely axiomatized. In contrast, H and all other gates are represented in $\Pi_{k}$ as combinatorial objects governed by a small, finite set of equations. These equations are both sound (every derivable equality holds in all models satisfying the axioms) and complete (every valid equality in such models is derivable). As a result, all reasoning, from the simplest identities to the most involved optimizations, proceeds without approximation, without special-purpose constants, and without leaving the symbolic framework.

For a simple example, let us prove the identity HH=id entirely within $\Pi_{k}$. In $\Pi_{k}$ with k≥3, define

$$\omega \;:=\;\zeta_{k}^{2^{k-3}}\text{,}\;S\;:=\;\text{id}⊕\omega^{2}\text{,}\;H\;:=\;\omega^{-1}\cdot \left(VSV\right)\text{.}$$

Starting from $H^{2}$:

$$\begin{array}{ccc} H^{2} & =(\omega^{-1}\cdot VSV)\;(\omega^{-1}\cdot VSV)\\ & =\omega^{-2}\cdot VSVVSV & \text{(centrality of scalars)}\\ & =\omega^{-2}\cdot V\;\left(SXS\right)\;V & (\text{by Axiom}\;[1]). \end{array}$$

Unfolding the definition $S=\text{id}⊕\omega^{2}$ and using the fact that the ⊕-swap respects ○ gives

$$\begin{array}{ccc} \mathrm{SXS} & =\left(\text{id}⊕\omega^{2}\right)\;X\;\left(\text{id}⊕\omega^{2}\right)\\ & =\left(\text{id}⊕\omega^{2}\right)\;\left(\omega^{2}⊕\text{id}\right)\;X & \\ & =(\omega^{2}⊕\omega^{2})\;X\\ & =\omega^{2}\cdot X & \text{(distributivity of scalars)}. \end{array}$$

Substituting back:

$$\begin{array}{ccc} H^{2} & =\omega^{-2}\cdot V\;\left(\omega^{2}\cdot X\right)\;V\\ & =VXV & \text{(centrality of scalars)}\\ & =V^{4} & \left(\text{by Axiom}\;[1]\right)\\ & =X^{2}\\ & =\text{id} & \text{(since}\;X^{2}=\sigma_{⊕}^{2}=\text{id}\text{)}. \end{array}$$

Thus, HH=id is derivable in $\Pi_{k}$ for all k≥3 using only the three axioms [**1**]–[**3**] and the axioms of bipermutative categories (which include all algebraic properties of scalars used, such as centrality and distributivity).

## Conclusion

We have provided an equational axiomatization of quantum computing and an accompanying free model, that does not use complex numbers and is entirely discrete and combinatorial in nature. The induced quantum programming language suffices to express any quantum computation (as stated in *Theorem* 3), and does not lose any universality or reasoning power with respect to the standard model. Complex linear algebra is not required, and in this universal model of quantum computing the full power of combinatorial optimization can be brought to bear on the problem of manipulating quantum programs in an automated way for optimization, specification, and verification of correctness. Whether two Boolean circuits are equivalent is an intractable problem in the worst case (46), but cases arising in practice can efficiently be dealt with heuristically (47). Whether two quantum circuits are equivalent is an even more intractable problem (13), as it additionally requires comparing whether two real numbers are equal. Because it is efficient to decide whether two elements of $D[\zeta_{2}]$ are equal, our free model can be dealt with efficiently with heuristics. Furthermore, the axiomatization is foundationally satisfactory, building on classical reversible computing, linking to various quantum computing hardware platforms, and isolating quantum advantage in the ability to take well-behaved square roots.

The literature mostly works with unitary groups $U_{2^{n}}\left(C\right)$ that are parametrized by the number n of qubits, but otherwise do not observe any relationships between individual groups in the family. Working with bipermutative categories instead means that there are no bounds on the completeness of the free model of *Theorem* 1 with respect to circuit size or gate count. More precisely, instead of having sets of equations that grow with, and depend on, the number of qubits or number of gates (48), the set of axioms [**1**]–[**3**] is complete for all circuits expressible in $\Pi_{k}$ at once.

## Supplementary Material

Appendix 01 (PDF)

## Data Availability

There are no data underlying this work.
