# Supplementary material for: Free quantum computing
Source: Proc Natl Acad Sci U S A. 2026 Feb 17;123(8):e2510881123. doi: 10.1073/pnas.2510881123 (PMC12933034; doi:10.1073/pnas.2510881123)
Supplement: Supplementary file 1 — Appendix 01 (PDF) [file pnas.2510881123.sapp.pdf]

Free Quantum Computing: Supporting Information

Jacques Carette[a], **Chris Heunen**[b,1], **Robin Kaarsgaard**[c], **Neil J. Ross**[d], **and Amr Sabry**[e]

[a]McMaster University, Department of Computing and Software, Canada; [b]University of Edinburgh, School of Informatics, United Kingdom; [c]University of Southern Denmark, Department of Mathematics and Computer Science, Denmark; [d]Dalhousie University, Department of Mathematics and Statistics, Canada; [e]Indiana University, Department of Compute Science, United States of America

This manuscript was compiled on January 15, 2026

DRAFT

This Supporting Information contains a proof that the free model of quantum computing described in the article is sound and complete (Theorem S30), and a proof of the claims about the asymptotic scaling of term size as precision varies. The former proof proceeds in three steps: first we reduce the situation to sums only, obviating the need to consider products; then we prove soundness and completeness in the reversible setting up to auxiliary qubits; and finally we establish soundness and completeness of the full model including measurement. We assume familiarity with categorical methods as used in quantum computing (1) thoughout.

First recall the definition of bipermutative categories (2, 3).

Definition S1. A *bipermutative category* is a category $\mathbf{C}$ with two strict symmetric monoidal structures $(\otimes, I)$ and $(\oplus, O)$ where

- for all objects A, B and all morphisms $f\colon A \to B$:

$$O \otimes A = O = A \otimes O$$
$$\mathrm{id}_O \otimes f = \mathrm{id}_O = f \otimes \mathrm{id}_O$$

- for all objects A, B, C and morphisms f, g, h:

$$(A \oplus B) \otimes C = (A \otimes C) \oplus (B \otimes C)$$
$$(f \oplus g) \otimes h = (f \otimes h) \oplus (g \otimes h)$$

  and the following diagram commutes:

$$\begin{array}{ccc} (A \oplus B) \otimes C & \xrightarrow{=} & (A \otimes C) \oplus (B \otimes C) \\ {\scriptstyle \sigma_\oplus \otimes \mathrm{id}}\downarrow & & \downarrow{\scriptstyle \sigma_\oplus} \\ (B \oplus A) \otimes C & \xrightarrow{=} & (B \otimes C) \oplus (A \otimes C) \end{array}$$

- for all objects A, B, C, D the diagram

$$\begin{array}{ccc} (A \oplus B) \otimes (C \oplus D) & \xrightarrow{=} & (A \otimes (C \oplus D)) \oplus (B \otimes (C \oplus D)) \\ {\scriptstyle \delta_L}\downarrow & & \downarrow{\scriptstyle \delta_L \oplus \delta_L} \\ ((A \oplus B) \otimes C) \oplus ((A \oplus B) \otimes D) & & (A \otimes C) \oplus (A \otimes D) \oplus (B \otimes C) \oplus (B \otimes D) \\ & \searrow^{=} & \downarrow{\scriptstyle \mathrm{id} \oplus \sigma_\oplus \oplus \mathrm{id}} \\ & & (A \otimes C) \oplus (B \otimes C) \oplus (A \otimes D) \oplus (B \otimes D) \end{array}$$

  commutes, where δ_L is the composite

$$A \otimes (B \oplus C) \xrightarrow{\sigma_\otimes} (B \oplus C) \otimes A \xrightarrow{=} (B \otimes A) \oplus (C \otimes A) \xrightarrow{\sigma_\otimes \oplus \sigma_\otimes} (A \otimes B) \oplus (A \otimes C).$$

A *strict bipermutative functor* is a functor that is strict symmetric monoidal for both $(\otimes, I)$ and $(\oplus, O)$.

A bipermutative category is *semisimple* when its objects are natural numbers, and its monoidal structures are $n \otimes m = nm$ (with unit 1) and $n \oplus m = n + m$ (with unit 0).

All our free models will be bipermutative categories, but some other models are weaker, such as the standard model of finite-dimensional C*-algebras and quantum channels. They are *rig categories*, where $(A \oplus B) \otimes C$ may not necessarily equal $(A \otimes C) \oplus (B \otimes C)$, but merely be isomorphic to it. No matter: any rig category is rig equivalent to a bipermutative category (2, 3).

In any monoidal category $(\mathbf{C}, \otimes, I)$ one can multiply any morphism $f\colon A \to B$ with a *scalar* $s\colon I \to I$ as $s \cdot f\colon A \to B$ as the following composite.

$$A \cong I \otimes A \xrightarrow{s \otimes f} I \otimes B \cong B$$

Tensor products

Notice that the axioms Eq. (1)–Eq. (3) only involve $\circ$ and $\oplus$, not $\otimes$. Moreover, the objects of Π_k are simply natural numbers. We will show that in every such category $\otimes$ can be reconstructed from $\oplus$. To be precise, we use *PROPs* (*PROduct and Permutation categories*) (4): strict symmetric monoidal categories $(\mathbf{C}, \oplus, 0)$ where objects are natural numbers and the sum of objects is addition of natural numbers. A *morphism of PROPs* is a strict symmetric monoidal functor between PROPs that is the identity on objects.

For natural numbers n and m, define a product $n \cdot m$ on objects in a PROP by the m-fold sum of n with itself, and a *Kronecker product* $f \otimes g$ of morphisms $f\colon m \to m'$ and $g\colon n \to n'$ by

$$mn \xrightarrow{n \cdot f} nm' \xrightarrow{\pi_{n,m'}} m'n \xrightarrow{m' \cdot g} m'n' \xrightarrow{\pi_{m',n'}} n'm'$$

where $n \cdot f = \overbrace{f \oplus \cdots \oplus f}^{n \text{ times}}$ and $\pi_{m,n}$ is the reindexing $mn \to nm$ that sends the lexicographic order on the ordinal number $m \times n$ to that on $n \times m$. Explicitly (5):

$$\pi_{m,n}(x) = \begin{cases} nx \bmod mn-1 & \text{if } x \neq mn-1, \\ mn-1 & \text{if } x = mn-1. \end{cases}$$

In general, there is no guarantee that $(f \otimes \mathrm{id}) \circ (\mathrm{id} \otimes g) = (\mathrm{id} \otimes g) \circ (f \otimes \mathrm{id})$. So a PROP need not induce a semisimple bipermutative category this way, but any semisimple bipermutative category *is* a PROP satisfying extra equations. To establish this claim, we will show that every morphism, even one ostensibly defined in terms of products, equals a morphism defined only in terms of sums. For objects this already holds by construction. For morphisms:

$$\begin{aligned} f \otimes g &= (f \otimes \mathrm{id}_n) \circ (\mathrm{id}_m \otimes g) \\ &= \sigma_\otimes \circ (\mathrm{id}_n \otimes f) \circ \sigma_\otimes \circ (\mathrm{id}_m \otimes g) \\ &= \sigma_\otimes \circ (\underbrace{f \oplus \cdots \oplus f}_{n \text{ times}}) \circ \sigma_\otimes \circ (\underbrace{g \oplus \cdots \oplus g}_{m \text{ times}}). \end{aligned}$$

By the three coherence morphisms in Theorem S1, it thus suffices to show that $\sigma_\otimes$ can always be expressed as a composition of sums of $\sigma_\oplus$; because δ_L is already derived from it by sums, and $\sigma_\oplus$ is already defined in terms of the sum.

Lemma S2. *In a semisimple bipermutative category, the multiplicative symmetry*

$$\sigma_\otimes\colon (A \oplus B) \otimes (C \oplus D) \to (C \oplus D) \otimes (A \oplus B)$$

can be expressed in terms of the symmetries $\sigma_\otimes$ on the strictly smaller objects $A \otimes (C \oplus D)$, $B \otimes (C \oplus D)$, $C \otimes (A \oplus B)$, $D \otimes (A \oplus B)$, $C \otimes A$, $D \otimes A$, $C \otimes B$, $D \otimes B$, and the additive symmetry $\sigma_\oplus$.

Proof. For the base case, observe that the symmetries $\sigma_\otimes\colon 1 \otimes n \to n \otimes 1$ and $\sigma_\otimes\colon n \otimes 1 \to 1 \otimes n$ are identity morphisms for any n. For $m > 1$, observe that the following diagram commutes:

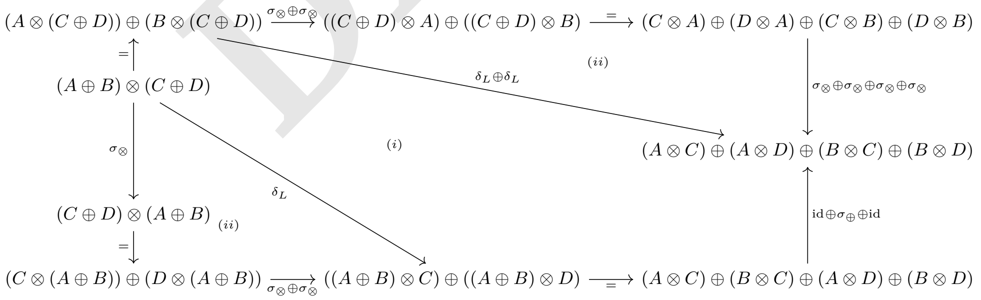

Indeed, (i) commutes by coherence condition (3, (IX)) and (ii) by definition of δ_L. Because $\sigma_\otimes\colon B \otimes A \to A \otimes B$ is the inverse of $\sigma_\otimes\colon A \otimes B \to B \otimes A$, it follows that the following diagram also commutes:

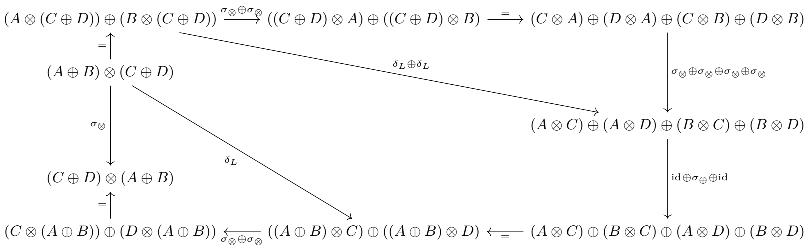

[1]

DRAFT

[1]E-mail: chris.heunen@ed.ac.uk

$\square$

Lemma S3. *In a semisimple bipermutative category, every multiplicative symmetry $\sigma_\otimes \colon m \otimes n \to n \otimes m$ can be constructed as a composition of sums of identities and additive symmetries $\sigma_\oplus$.*

Proof. We proceed by well-founded induction on the pair $[m, n]$ under lexicographic ordering. Write $\sigma_{\otimes[m,n]}$ for the multiplicative symmetry $m \otimes n \to n \otimes m$.

The base case $m = n = 0$ is vacuous because then $\sigma_\otimes = \mathrm{id}_0$. For the induction step, assume that $\sigma_{\otimes[m',n']}$ is a composition of sums of identities and additive symmetries $\sigma_\oplus$ for all (m', n') lexicographically before (m, n). By diagram Eq. (1) in the proof above, $\sigma_{\otimes[m,n]}$ is a composition of sums of identities and $\sigma_{\otimes[n-1,m]}$, $\sigma_{\otimes[1,m]}$, $\sigma_{\otimes[n-1,m-1]}$, $\sigma_{\otimes[n-1,1]}$, $\sigma_{\otimes[1,m-1]}$, $\sigma_{\otimes[1,1]}$, $\sigma_{\otimes[m-1,n]}$, and $\sigma_{\otimes[n,1]}$, as well as $\sigma_\oplus$. All these indices are lexicographically before $[n, m]$, except $[m-1, n]$. So for all multiplicative symmetries except $\sigma_{\otimes[m-1,n]}$, we can apply the induction hypothesis to write them as compositions of sums of identities and additive symmetries.

To construct $\sigma_{\otimes[m-1,n]}$, notice that $\sigma^{-1}_{\otimes[m-1,n]} = \sigma_{\otimes[n,m-1]}$. Apply induction to construct $\sigma_{\otimes[n,m-1]}$ as a composition of sums of identities and additive symmetries. As the inverse of identities and additive symmetries are, again, identities and additive summetries (respectively), this immediately lets us construct $\sigma_{\otimes[m-1,n]}$ of the right form. $\square$

Theorem S4. *Semisimple bipermutative categories are PROPs, and a functor between semisimple bipermutative categories is a strict bipermutative functor if and only if it is a PROP morphism.*

Proof. By definition, the objects of semisimple bipermutative categories are precisely natural numbers. As regards morphisms, by distributivity every monoidal product of morphisms reduces to monoidal sums and multiplicative symmetries, and by Theorem S3 every coherence isomorphism of semisimple bipermutative categories – and, consequently, every coherence condition of bipermutative categories – can be expressed in terms of the strict symmetric monoidal structure $(\oplus, 0)$.

If $F \colon \mathbf{C} \to \mathbf{D}$ is a functor between semisimple bipermutative categories, it follows immediately that if F is a strict bipermutative functor it also a PROP morphism. Conversely, suppose F is a PROP morphism. Since $F(n) = n$ on morphisms in particular $F(1) = 1$, so on objects it preserves the multiplicative unit. On morphisms it follows from Theorem S3 that $F(\sigma_\otimes) = \sigma_\otimes$ since $\sigma_\otimes$ is constructed (in both $\mathbf{C}$ and $\mathbf{D}$) purely in terms of the strict symmetric monoidal structure $(\oplus, 0)$, which is preserved exactly by F. Moreover, F preserves monoidal products strictly:

$$
\begin{aligned}
& F(f \otimes g) \\
&= F((f \otimes \mathrm{id}_n) \circ (\mathrm{id}_m \otimes g)) \\
&= F(\sigma_\otimes \circ (\mathrm{id}_n \otimes f) \circ \sigma_\otimes \circ (\mathrm{id}_m \otimes g)) \\
&= F(\sigma_\otimes \circ (\underbrace{f \oplus f \oplus \cdots \oplus f}_{n \text{ times}}) \circ \sigma_\otimes \circ (\underbrace{g \oplus g \oplus \cdots \oplus g}_{m \text{ times}}) \\
&= F(\sigma_\otimes) \circ F(\underbrace{f \oplus f \oplus \cdots \oplus f}_{n \text{ times}}) \circ F(\sigma_\otimes) \circ F(\underbrace{g \oplus g \oplus \cdots \oplus g}_{m \text{ times}}) \\
&= F(\sigma_\otimes) \circ \underbrace{F(f) \oplus F(f) \oplus \cdots \oplus F(f)}_{n \text{ times}} \circ F(\sigma_\otimes) \circ \underbrace{F(g) \oplus F(g) \oplus \cdots \oplus F(g)}_{m \text{ times}} \\
&= \sigma_\otimes \circ \underbrace{F(f) \oplus F(f) \oplus \cdots \oplus F(f)}_{n \text{ times}} \circ \sigma_\otimes \circ \underbrace{F(g) \oplus F(g) \oplus \cdots \oplus F(g)}_{m \text{ times}} \\
&= \sigma_\otimes \circ (\mathrm{id}_n \otimes F(f)) \circ \sigma_\otimes \circ (\mathrm{id}_m \otimes F(g)) \\
&= (F(f) \otimes \mathrm{id}_n) \circ (\mathrm{id}_m \otimes F(g)) \\
&= F(f) \otimes F(g).
\end{aligned}
$$

Thus F is a strict symmetric monoidal functor for $(\otimes, 1)$ as well, in turn making it a strict bipermutative functor. $\square$

Because free PROPs are known to exist (4), this proves Theorem 1 of the main article.

Proposition S5. *There exists a free bipermutative category $\langle \zeta_k, S, V \rangle$ on generators $\zeta_k \colon 1 \to 1$ and $V \colon 1 \oplus 1 \to 1 \oplus 1$ for $k \geq 2$. Write X for the symmetry morphism $\sigma \colon 1 \oplus 1 \to 1 \oplus 1$, and S for $1 \oplus \zeta_k^{2^{k-2}} \colon 1 \oplus 1 \to 1 \oplus 1$. There exists a quotient Π_k of $\langle \zeta_k, S, V \rangle$ by the smallest congruence $\sim_k$ of bipermutative categories containing equations* Eq. (1)–Eq. (3)*, and it is a bipermutative category.*

Cancellativity

Note that throughout this section, we use a, a', b, b', c to denote morphisms of a category $\mathbf{C}$.

Next, we build towards soundness and completeness of Π_k. Our proof will use auxiliary objects by relying on the following property.

DRAFT

Definition S6. A monoidal category $(\mathbf{C}, \oplus, O)$ is *strongly cancellative* when

$$a \oplus b = a' \oplus b' \implies a = a' \text{ and } b = b'$$

for all morphisms $a, a' \colon A \to A'$ and $b, b' \colon B \to B'$.

Any monoidal category that embeds into one where $\oplus$ is a biproduct is strongly cancellative. This includes the PROP $\mathbf{Unitary}(R)$ of unitary matrices over an involutive ring R, which embeds into the rig category of R-modules, where $a \oplus b$ is a block-diagonal unitary matrix. We believe that Π_k itself is strongly cancellative for all k (it is known to be the case for $k = 2$), but leave a proof of this as an open question. All we need here is to force a PROP to become strongly cancellative.

Proposition S7. *Any symmetric monoidal category $(\mathbf{C}, \oplus, O)$ has a monoidal congruence defined for $a, a' \colon A \to A'$ by:*

$$a \approx a' \qquad \iff \qquad \exists b, b' \colon B \to B' \colon a \oplus b = a' \oplus b'$$

Proof. We verify that the relation $\approx$ is a monoidal congruence:

- Reflexivity. Taking $b = b' = \mathrm{id}_I$ shows that $a \approx a$.
- Symmetry. Suppose that $a \approx a'$, say because $a \oplus b = a' \oplus b'$. Then also $a' \oplus b' = a \oplus b$, so $a' \approx a$.
- Transivity. Suppose that $a \approx a'$ because $a \oplus b = a' \oplus b'$, and that $a' \approx a''$ because $a' \oplus c' = a'' \oplus c''$. Then $a \approx a''$ because

$$\begin{aligned} a \oplus (c' \circ b) &= (1 \oplus c') \circ (a \oplus b) \\ &= (1 \oplus c') \circ (a' \oplus b') \\ &= (a' \oplus c') \circ (1 \oplus b') \\ &= (a'' \oplus c'') \circ (1 \oplus b') \\ &= a'' \oplus (c'' \circ b'). \end{aligned}$$

- Compositionality. Suppose that $a \approx a'$ because $a \oplus b = a' \oplus b'$ for $a, a' \colon A \to A'$, and that $c \approx c'$ because $c \oplus d = c' \oplus d'$ for $c, c' \colon A' \to A''$. Then $ca \approx c'a'$ because

$$ca \oplus db = (c \oplus d)(a \oplus b) = (c' \oplus d')(a' \oplus b') = c'a' \oplus d'b'.$$

- Monoidality. Suppose that $a \approx a'$ because $a \oplus b = a' \oplus b'$, and that $c \approx c'$ because $c \oplus d = c' \oplus d'$. Then

$$a \oplus b \oplus c \oplus d = a' \oplus b' \oplus c' \oplus d'$$

  and by postcomposing with the symmetry hence also

$$a \oplus c \oplus b \oplus d = a' \oplus c' \oplus b' \oplus d',$$

  that is, $a \oplus c \approx a' \oplus c'$.

□

DRAFT

Corollary S8. *If $(\mathbf{C}, \oplus, O)$ is a symmetric monoidal category, then $\mathbf{C}/\approx$ is a well-defined strongly cancellative symmetric monoidal category. If $\mathbf{C}$ satisfies some equation, then so does $\mathbf{C}/\approx$.*

Proof. The first statement follows directly from Theorem S7. The second statement follows from the fact that the (symmetric) monoidal functor $\mathbf{C} \to \mathbf{C}/\approx$ preserves equations. □

We can now define the functor of Theorem 2 and Theorem 3 of the main article.

Proposition S9. *The following defines a symmetric monoidal functor $[\![-]\!] \colon \Pi_k \to \mathbf{Unitary}(\mathbb{D}[\zeta_k])$:*

$$[\![\zeta_k]\!] = e^{2\pi i/2^k} \qquad [\![V]\!] = \frac{1}{2}\begin{pmatrix} 1+i & 1-i \\ 1-i & 1+i \end{pmatrix}$$

Proof. Observe that these matrices satisfy Eq. (1)–Eq. (3). □

Because $\mathbf{Unitary}(\mathbb{D}[\zeta_k])$ is strongly cancellative, there is also a symmetric monoidal functor $\langle \zeta_k, S, V \rangle \to \mathbf{Unitary}(\mathbb{D}[\zeta_k])$.

Definition S10. For $k \geq 2$, define the following standard morphisms in Π_k:

$$-1 = \zeta_k^{2^{k-1}} \colon 1 \to 1 \qquad\qquad X = \sigma_\oplus \colon 2 \to 2$$

$$H = (\zeta_k^{2^{k-3}})^7 \bullet T_k^{2^{k-2}} V T_k^{2^{k-2}} \colon 2 \to 2 \;\; (k \geq 3) \qquad\qquad T_k = \mathrm{id} \oplus \zeta_k \colon 2 \to 2$$

The ring $\mathbb{D}[\zeta_k]$ has a conjugation $x + \zeta_k y \mapsto (x + \zeta_k y)^* = x - \zeta_k y$, and this ring automorphism extends componentwise to an automorphism on the ring of matrices with entries in $\mathbb{D}[\zeta_k]$. We now define a syntactic analogue.

Definition S11. Let $a \in \Pi_k$ for $k \geq 2$. The *conjugate* a^* of a is defined inductively by:

- $(\mathrm{id}_n)^* = \mathrm{id}_n$;
- $(\sigma)^* = \sigma$;
- $(\zeta_k)^* = -\zeta_k$;
- $(V)^* = V$;
- $(ab)^* = (a)^*(b)^*$, for any $a, b \colon m \to m$;
- $(a \oplus b)^* = (a)^* \oplus (b)^*$, for any $a \colon m \to m$ and $b \colon n \to n$.

A straightforward induction shows that $[\![a^*]\!] = [\![a]\!]^*$.

Definition S12. Let $k \geq 3$. The *catalytic embedding* $\Phi_k \colon \Pi_k \to \Pi_{k-1}$ is the symmetric monoidal functor defined on objects by $\Phi_k(n) = 2n$ and on morphisms by

- $\Phi_k(\mathrm{id}_n) = \mathrm{id}_n \oplus \mathrm{id}_n$;
- $\Phi_k(\sigma) = \sigma \oplus \sigma$;
- $\Phi_k(\zeta_k) = X \circ (\mathrm{id} \oplus \zeta_{k-1})$;
- $\Phi_k(V) = V \oplus V$;
- $\Phi_k(ab) = \Phi_k(a)\Phi_k(b)$ for $a, b \colon n \to n$;
- $\Phi_k(a \oplus b) = \sigma^{\otimes}_{2,m+n} \Phi_k(a) \oplus \Phi_k(b) \sigma^{\otimes}_{2,m+n}$, for $a \colon m \to m$ and $b \colon n \to n$.

For $n \in \mathbb{N}$, the *catalyst* $c_{k,n} \colon 2n \to 2n$ is $c_{k,n} = (H \circ T_k) \otimes \mathrm{id}_n$.

Lemma S13. *If $a \colon n \to n$ in Π_k for $k \geq 3$, then $c_{k,n} \Phi_k(a) c^{\dagger}_{k,n} \sim_k a \oplus a^*$.*

Proof. Proceed by structural induction on a. If $a = \mathrm{id}_n$, then

$$
\begin{aligned}
c_{k,n} \circ \Phi_k(a) \circ c^{\dagger}_{k,n} &\sim_k c_{k,n} \circ \Phi_k(\mathrm{id}_n) \circ c^{\dagger}_{k,n} \\
&\sim_k (HT \otimes \mathrm{id}_n) \circ \mathrm{id}_n \oplus \mathrm{id}_n \circ (T^{\dagger} H^{\dagger} \otimes \mathrm{id}_n) \\
&\sim_k (HT \otimes \mathrm{id}_n) \circ \mathrm{id}_2 \otimes \mathrm{id}_n \circ (T^{\dagger} H^{\dagger} \otimes \mathrm{id}_n) \\
&\sim_k (HT \circ \mathrm{id}_2 \circ T^{\dagger} H^{\dagger}) \otimes \mathrm{id}_n \\
&\sim_k \mathrm{id}_2 \otimes \mathrm{id}_n \\
&\sim_k \mathrm{id}_n \oplus \mathrm{id}_n \\
&\sim_k a \oplus a^*.
\end{aligned}
$$

If $a = \sigma_{m,n}$, then

$$
\begin{aligned}
c_{k,m+n} \circ \Phi_k(a) \circ c^{\dagger}_{k,m+n} &\sim_k c_{k,m+n} \circ \Phi_k(\sigma_{m,n}) \circ c^{\dagger}_{k,m+n} \\
&\sim_k (HT \otimes \mathrm{id}_{m+n}) \circ \sigma_{m,n} \oplus \sigma_{m,n} \circ (T^{\dagger} H^{\dagger} \otimes \mathrm{id}_{m+n}) \\
&\sim_k (HT \otimes \mathrm{id}_{m+n}) \circ \mathrm{id}_2 \otimes \sigma_{m,n} \circ (T^{\dagger} H^{\dagger} \otimes \mathrm{id}_{m+n}) \\
&\sim_k (HT \circ \mathrm{id}_2 \circ T^{\dagger} H^{\dagger}) \otimes \sigma_{m,n} \\
&\sim_k \mathrm{id}_2 \otimes \sigma_{m,n} \\
&\sim_k \sigma_{m,n} \oplus \sigma_{m,n} \\
&\sim_k a \oplus a^*
\end{aligned}
$$

DRAFT

If $a = \zeta_k$, then

$$\begin{aligned}
c_{k,1} \circ \Phi_k(a) \circ c_{k,1}^\dagger &\sim_k c_{k,1} \circ \Phi_k(\zeta_k) \circ c_{k,1}^\dagger \\
&\sim_k (HT \otimes \mathrm{id}_1) \circ (X \circ (\mathrm{id} \oplus \zeta_{k-1})) \circ (T^\dagger H^\dagger \otimes \mathrm{id}_1) \\
&\sim_k HT \circ (X \circ (\mathrm{id} \oplus \zeta_{k-1})) \circ T^\dagger H^\dagger \\
&\sim_k H \circ T \circ X \circ T^2 \circ T^\dagger \circ H^\dagger \\
&\sim_k H \circ T \circ X \circ T \circ H^\dagger \\
&\sim_k H \circ (\zeta_k \oplus \zeta_k) \circ X \circ H^\dagger \\
&\sim_k (\zeta_k \oplus \zeta_k) \circ Z \\
&\sim_k \zeta_k \oplus -\zeta_k \\
&\sim_k a \oplus a^*
\end{aligned}$$

If $a = V$, then

$$\begin{aligned}
c_{k,2} \circ \Phi_k(a) \circ c_{k,2}^\dagger &\sim_k c_{k,2} \circ \Phi_k(V) \circ c_{k,2}^\dagger \\
&\sim_k (HT \otimes \mathrm{id}_2) \circ V \oplus V \circ (T^\dagger H^\dagger \otimes \mathrm{id}_2) \\
&\sim_k (HT \otimes \mathrm{id}_2) \circ \mathrm{id}_2 \otimes V \circ (T^\dagger H^\dagger \otimes \mathrm{id}_2) \\
&\sim_k (HT \circ \mathrm{id}_2 \circ T^\dagger H^\dagger) \otimes V \\
&\sim_k \mathrm{id}_2 \otimes V \\
&\sim_k V \oplus V \\
&\sim_k a \oplus a^*
\end{aligned}$$

If $a = a_1 a_2$, for $a_1, a_2 \colon m \to m$ in Π_k, then by induction,

$$\begin{aligned}
c_{k,m} \circ \Phi_k(a) \circ c_{k,m}^\dagger &\sim_k c_{k,m} \circ \Phi_k(a_1 a_2) \circ c_{k,m}^\dagger \\
&\sim_k c_{k,m} \circ \Phi_k(a_1)\Phi_k(a_2) \circ c_k^\dagger \\
&\sim_k c_{k,m} \circ \Phi_k(a_1) \circ c_{k,m} \circ c_{k,m}^\dagger \Phi_k(a_2) \circ c_k^\dagger \\
&\sim_k a_1 \oplus a_1^* \circ a_2 \oplus a_2^* \\
&\sim_k (a_1 a_2) \oplus (a_1^* a_2^*) \\
&\sim_k a \oplus a^*
\end{aligned}$$

Finally, consider the case $a = a_1 \oplus a_2$, for $a_1 \colon m \to m$ and $a_2 \colon n \to n$ in Π_k. Observe

$$\begin{aligned}
c_{k,m+n} \circ \sigma_{2,m+n}^{\otimes} &\sim_k (HT \otimes \mathrm{id}_{m+n}) \circ \sigma_{2,m+n}^{\otimes} \\
&\sim_k \sigma_{2,m+n}^{\otimes} \circ (\mathrm{id}_{m+n} \otimes HT) \\
&\sim_k \sigma_{2,m+n}^{\otimes} \circ ((\mathrm{id}_m \oplus \mathrm{id}_n) \otimes HT) \\
&\sim_k \sigma_{2,m+n}^{\otimes} \circ ((\mathrm{id}_m \otimes HT) \oplus (\mathrm{id}_n \otimes HT)) \\
&\sim_k \sigma_{2,m+n}^{\otimes} \circ (c_{k,m} \oplus c_{k,n}).
\end{aligned}$$

Hence, by induction,

$$\begin{aligned}
c_{k,m+n} \circ \Phi_k(a) \circ c_{k,m+n}^\dagger &\sim_k c_{k,m+n} \circ \Phi_k(a_1 \oplus a_2) \circ c_{k,m+n}^\dagger \\
&\sim_k c_{k,m+n} \circ (\sigma_{2,m+n}^{\otimes} \Phi_k(a) \oplus \Phi_k(b) \sigma_{2,m+n}^{\otimes}) \circ c_{k,m+n}^\dagger \\
&\sim_k \sigma_{2,m+n}^{\otimes} (c_{k,m} \oplus c_{k,n}) \Phi_k(a) \oplus \Phi_k(b) (c_{k,m}^\dagger \oplus c_{k,n}^\dagger) \sigma_{2,m+n}^{\otimes} \\
&\sim_k \sigma_{2,m+n}^{\otimes} (a_1 \oplus a_1^*) \oplus (a_2 \oplus a_2^*) \sigma_{2,m+n}^{\otimes} \\
&\sim_k (a_1 \oplus a_2) \oplus (a_1^* \oplus a_2^*) \\
&\sim_k a \oplus a^*.
\end{aligned}$$

□

We can now prove Theorem 4 from the main article.

Lemma S14. *If $k \geq 3$, and $a, b \in \Pi_k$ satisfy $[\![a]\!] = [\![b]\!]$, then $[\![\Phi_k(a)]\!] = [\![\Phi_k(b)]\!]$.*

DRAFT

Proof. Let $a, b \in \Pi_k$ and suppose that $[\![a]\!] = [\![b]\!]$, By Theorem S13,

$$c_k \Phi_k(a) c_k^\dagger \sim_k a \oplus a^* \quad \text{and} \quad c_k \Phi_k(b) c_k^\dagger \sim_k b \oplus b^*.$$

Because the axioms are sound with respect to the interpretation, then

$$\begin{aligned}
[\![c_k]\!][\![\Phi_k(a)]\!][\![c_k^\dagger]\!] &= [\![c_k \Phi_k(a) c_k^\dagger]\!] \\
&= [\![a \oplus a^*]\!] \\
&= [\![a]\!] \oplus [\![a]\!]^* \\
&= [\![b]\!] \oplus [\![b]\!]^* \\
&= [\![b \oplus b^*]\!] \\
&= [\![c_k \Phi_k(b) c_k^\dagger]\!].
\end{aligned}$$

We conclude that

$$[\![\Phi_k(a)]\!] = [\![c_k^\dagger]\!][\![c_k]\!][\![\Phi_k(a)]\!][\![c_k^\dagger]\!][\![c_k]\!] = [\![c_k^\dagger]\!][\![c_k]\!][\![\Phi_k(b)]\!][\![c_k^\dagger]\!][\![c_k]\!] = [\![\Phi_k(b)]\!].$$ □

Write $\approx_k$ for the smallest congruence of bipermutative categories containing $\sim_k$ and $\approx$. It is clear that if $a \approx_k b$ for $a, b \in \Pi_k$, then $[\![a]\!] = [\![b]\!]$, that is, the equations of Π_k are *sound*. The following theorem shows *completeness*, which is the converse.

Theorem S15. *Let $k \geq 2$. If $a, b \in \Pi_k$ satisfy $[\![a]\!] = [\![b]\!]$, then $a \approx_k b$.*

Proof. The proof proceeds by induction on k. The base case $k = 2$ is known (6). Now assume that the property holds for k, and suppose that $a, b \in \Pi_{k+1}$ satisfy $[\![a]\!] = [\![b]\!]$. Then $\Phi_k(a), \Phi_k(b) \in \Pi_k$ and, by Theorem S14, $[\![\Phi_k(a)]\!] = [\![\Phi_k(b)]\!]$. Therefore, by the induction hypothesis, $\Phi_k(a) \approx_k \Phi_k(b)$. Hence, $\Phi_k(a) \approx_{k+1} \Phi_{k+1}(b)$, and thus $C\Phi_k(a)C^\dagger \approx_{k+1} C\Phi_{k+1}(b)C^\dagger$. Theorem S13 now provides $a', b' \in \Pi_{k+1}$ satisfying

$$a \oplus a' \approx_{k+1} C\Phi_{k+1}(a)C^\dagger \approx_{k+1} C\Phi_{k+1}(b)C^\dagger \approx_{k+1} b \oplus b'.$$

Thus $a \approx_{k+1} b$ by strong cancellation. □

DRAFT

The goal of the rest of this Supporting Information is to prove completeness, in Theorem S30 below, of the free model including measurement. The main idea is to reduce to an existing algebraic axiomatisation (7). This is achieved in four stages. After defining the free model of measurement, we make precise how a model can validate equations. For this to make sense, we then show that the free model including measurement supports classical control, by first reducing to manipulations of injections between finite sets. Finally, we can then verify the required axioms.

Measurement can be freely added to any model in three steps, as follows (8, 9). Let $\mathbf{C}$ be a rig category.

1. Freely add the ability to initialise states. This results in a category $R(\mathbf{C})$ that has the same objects as $\mathbf{C}$, but morphisms $A \to B$ in $R(\mathbf{C})$ are morphisms $A \oplus H \to B$ in $\mathbf{C}$ for some *heap* object H, where morphisms are identified when they are equal up to preprocessing the heap. There is a unique morphism $O \to A$ in $R(\mathbf{C})$ for each object A, that initialises states. If $\mathbf{C}$ is the PROP of unitary matrices, then $R(\mathbf{C})$ is the category of isometries.

2. Freely add decoherence. This results in a category $\mathrm{LR}(\mathbf{C})$ that has the same objects as $\mathbf{C}$, but morphisms $A \to B$ in $\mathrm{LR}(\mathbf{C})$ are morphisms $A \to B \otimes G$ in $R(\mathbf{C})$ for some *garbage* object G, where morphisms are identified when they are equal up to postprocessing the garbage. There is a unique morphism $A \to I$ in $\mathrm{LR}(\mathbf{C})$ for each object A, that discards the system A by removing it from the control of the experimenter to the environment, according to Zurek's interpretation of decoherence (10). If $\mathbf{C}$ is the PROP of unitary matrices, then $\mathrm{LR}(\mathbf{C})$ is the category of finite-dimensional Hilbert spaces and completely positive linear maps, also known as quantum channels.

3. Freely add classical control, *i.e.* the ability to branch on measurement outcomes. This results in a category $\mathrm{Split}(\mathrm{LR}(\mathbf{C}))$, where objects are projections $p^\dagger = p^2 = p \colon A \to A$ in $\mathrm{LR}(\mathbf{C})$, and where morphisms $p \to p'$ are $f \colon A \to A'$ in $\mathrm{LR}(\mathbf{C})$ satisfying $p' f p = f$. This is also known as the *Cauchy completion* or *Karoubi envelope* (11), and has the universal property that projections on B in $\mathrm{LR}(\mathbf{C})$ correspond to embeddings $A \to B$ in $\mathrm{Split}(\mathrm{LR}(\mathbf{C}))$. If $\mathbf{C}$ is the PROP of unitary matrices, then $\mathrm{Split}(\mathrm{LR}(\mathbf{C}))$ is the category of finite-dimensional C*-algebras and quantum channels.

The $\oplus$ of the original rig category $\mathbf{C}$ becomes a coproduct in $\mathrm{Split}(\mathrm{LR}(\mathbf{C}))$, and for this to happen there is no choice in the order in which these steps are taken (8, 9).

Algebraic theories

An important step in the axiomatisation of quantum computation is Staton's algebraic theory of quantum computation (7). This shows that the theory of finite-dimensional C*-algebras and quantum channels can be reduced to a small number of generating spaces, channels, and equations between them. In brief, an algebraic theory consists of two things:

- Some *operations*, each carrying an arity $p \to (n_1, \dots, n_m)$, meaning that the operation accepts p parameters and produces a number of results in the list $n_1, \dots, n_m$. Operations can be combined both in sequence (when the number of parameters match) and in parallel to form *terms* t, which describe computations.
- Some *equations* describing which terms are equal.

As the name suggests, this setup is entirely analogous to that found in algebra – for example in combinatorial group theory, where groups are described in terms of a number of elementary operations (*generators*) and equations between elements of the group (*relations*).

The algebraic theory of quantum computation consists of three operations (and twelve equations, which we disregard for now). The operations are:

- $\mathsf{new}\colon 0 \to 1$ which allocates a new qubit in the state $|0\rangle$.
- $\mathsf{measure}\colon 1 \to (0,0)$ which measures a qubit in the computational basis and records the outcome in the choice of output.
- $\mathsf{apply}_U : n \to n$ which applies an n-qubit unitary U.

An algebraic theory can be interpreted in a category that has enough structure to support the operations and equations:

1. It must have coproducts, it must have a distinguished object X, and for each operation $O\colon n \to n_1, \dots, n_m$ a morphism $[\![O]\!]\colon n \bullet X \to (n_1 \bullet X + \cdots + n_m \bullet X)$ for an *action* $n \bullet -$ of the PROP **FinPerm** whose morphisms $n \to n$ are the permutations of $\{1, \dots, n\}$ (and there are no morphisms $n \to n'$ if $n \neq n'$) (12).
2. For each equation $s = t$, it must be the case that the morphisms $[\![s]\!]$ and $[\![t]\!]$ coincide: $[\![s]\!] = [\![t]\!]$.

Such a category is called a *model* of the algebraic theory.

The algebraic theory of quantum computation has a natural model given by the category of finite-dimensional C*-algebras and quantum channels. Here, a term t of arity $(p \mid n_1, \dots, n_m)$ describes a quantum channel $[\![t]\!]$ from the algebra $M_p(\mathbb{C})$ of 2^p-by-2^p matrices to the direct sum of matrix algebras $M_{n_1}(\mathbb{C}) \oplus \cdots \oplus M_{n_m}(\mathbb{C})$. Equations $t = u$ are assertions of the fact that the quantum channels $[\![t]\!]$ and $[\![u]\!]$ do the same thing, i.e., $[\![t]\!] = [\![u]\!]$. What is surprising is that this model is *complete*: if $[\![t]\!] = [\![u]\!]$ happens to be the case (for any choice of quantum channel t and u), then this can be established purely by applying the equations of the algebraic theory.

Our goal is to prove that $\mathrm{Split}(\mathrm{LR}(\Pi_k))$ is also a complete model of Staton's algebraic theory of quantum computation. For that to make sense, we first explain in full detail how categories can interpret algebraic theories.

For a category $\mathbf{C}$ to host a structure for the signature of an algebraic theory, it must have an *action* for **FinPerm**: there must be a functor $\bullet\colon \mathbf{FinPerm} \times \mathbf{C} \to \mathbf{C}$ and natural isomorphisms $0 \bullet X \cong X$ and $(n+m) \bullet X \cong n \bullet (m \bullet X)$ subject to certain coherence conditions. We start by showing that any symmetric monoidal category $\mathbf{C}$ gives rise to a family of canonical actions for **FinPerm**.

Let $A^{\otimes n}$ denote the n-fold monoidal product of an object A with itself (choosing a canonical bracketing, e.g., leaning all parentheses to the left), with $A^{\otimes 0} = I$. Define the *exponential action* of an object S in $\mathbf{C}$ to be the functor $\bullet\colon \mathbf{FinPerm} \times \mathbf{C} \to \mathbf{C}$ given by $n \bullet X = S^{\otimes n} \otimes X$ on objects, and on morphisms $(\pi : n \to n) \bullet (f\colon X \to Y) = \lceil \pi \rceil \otimes f$, where $\lceil \pi \rceil\colon S^{\otimes n} \to S^{\otimes n}$ is the unique permutation that uses the natural symmetry $\sigma_{X,Y}\colon X \otimes Y \to Y \otimes X$ to permute the n different S-wires according to the permutation $\pi\colon n \to n$.

Lemma S16. *When* $(\mathbf{C}, \otimes, I)$ *is a symmetric monoidal category and* S *is an object of* $\mathbf{C}$*, the* exponential action of S on $\otimes$ *is an action of* **FinPerm** *on* $\mathbf{C}$*.*

Proof. To see that $\bullet : \mathbf{FinPerm} \times \mathbf{C} \to \mathbf{C}$ is bifunctorial, we notice that

$$\mathrm{id}_n \bullet \mathrm{id}_X = \lceil \mathrm{id}_n \rceil \otimes \mathrm{id}_X = \mathrm{id}_{S^{\otimes n}} \otimes \mathrm{id}_X = \mathrm{id}_{S^{\otimes n} \otimes X} = \mathrm{id}_{n \bullet X}$$

and

$$\begin{aligned}(\pi_2 \bullet g) \circ (\pi_1 \bullet f) &= (\lceil \pi_2 \rceil \otimes g) \circ (\lceil \pi_1 \rceil \otimes f) \\ &= (\lceil \pi_2 \rceil \circ \lceil \pi_1 \rceil) \otimes (g \circ f) \\ &= \lceil \pi_2 \circ \pi_1 \rceil \otimes (g \circ f) \\ &= (\pi_2 \circ \pi_1) \bullet (g \circ f)\end{aligned}$$

using the fact that permuting n wires according to first π_1 and then π_2 is exactly the same as permuting them according to their composition $\pi_2 \circ \pi_1$. To see that this is an action, we notice first that

$$0 \bullet X = S^{\otimes 0} \otimes X = I \otimes X \cong X$$

DRAFT

and

$$\begin{aligned}(n+m)\bullet X &= S^{\otimes n+m}\otimes X\\ &\cong S^{\otimes n}\otimes (S^{\otimes m}\otimes X)\\ &= n\bullet (m\bullet X)\end{aligned}$$

where the isomorphism $S^{\otimes n+m}\otimes X \cong S^{\otimes n}\otimes(S^{\otimes m}\otimes X)$ uses associativity to move parentheses accordingly. As for the coherence conditions, we may freely assume that $\mathbf{C}$ is strict symmetric monoidal, and notice that **FinPerm** already is strict symmetric monoidal. In this strict case, we notice that the isomorphisms $\vartheta : (n+m)\bullet X \to n\bullet(m\bullet X)$ and $\nu : 0\bullet X \to X$ given above, as well as all coherence isomorphisms of $\mathbf{C}$ and **FinPerm**, are identities, so the three coherence conditions (12) trivially commute (by virtue of every morphism involved being the identity). □

We are now ready to define structures for a signature. Instead of the Heisenberg picture (7), we will use the Schrödinger picture. For the following definition to make sense, there must be coproducts, which we will avail ourselves in theorem S25 below.

Definition S17. A *signature* is a set of operations with arity $O\colon p \to (m_1,\dots,m_k)$, intuitively specifying that operation O takes p many inputs and can produce either m_1, m_2, …, or m_k many outputs. Let $\mathbf{C}$ have an action of **FinPerm**. A *structure* for a signature in $\mathbf{C}$ is an object X together with, for each operation $O\colon p \to (m_1,\dots,m_k)$ a morphism $p\bullet X \to (m_1\bullet X)+\cdots+(m_k\bullet X)$.

The free coaffine rig category

Many of the equations we will be interested in are easily seen to hold in the PROP **FinInj** whose morphisms $m\to n$ are injections $\{1,\dots,m\}\rightarrowtail\{1,\dots,n\}$. The rig structure is given on objects by addition and multiplication of natural numbers. On morphisms $f\colon n\to m$ and $g\colon p\to q$, their sum $f\oplus g\colon n+p\to m+q$ is

$$(f\oplus g)(a) = \begin{cases} f(a) & \text{if } a\le n\\ m+g(a-n) & \text{otherwise}\end{cases}$$

while their product $f\otimes g\colon np\to mq$ is given by the function

$$\begin{aligned} p_{f,g}\colon n\times p &\to m\times q\\ (a,b)&\mapsto (f(a),g(b))\end{aligned}$$

conjugated by the Cantor permutation $\{1,\dots,np\}\to\{1,\dots,n\}\times\{1,\dots,p\}$.

Lemma S18. *R(**FinPerm**) is rig isomorphic to* **FinInj**.

Proof. It follows by the universal property of the R-construction that the forgetful functor $F\colon$ **FinPerm** $\to$ **FinInj** factors as

$$\begin{array}{ccc} \mathbf{FinPerm} & \xrightarrow{\mathcal{E}} & R(\mathbf{FinPerm})\\ & {\scriptstyle F}\searrow & \downarrow{\scriptstyle \hat{F}}\\ & & \mathbf{FinInj}\end{array}$$

for a unique rig functor $\hat{F}$. Since F and $\mathcal{E}$ are both identity on objects, it follows that $\hat{F}$ is identity (so specifically bijective) on objects as well: as such, $\hat{F}$ is an isomorphism of rig categories iff it is full and faithful.

By the Expansion-Raw Morphism factorisation (13, Lemma 2.8), every morphism in R(**FinPerm**) factors uniquely as a canonical injection $\amalg_1 : n\to n+k$ followed by a bijection $\pi\colon n+k\to m$. It follows that $\hat{F}$ is full iff every injection between finite sets admits such a factorisation, and faithful iff this factorisation is unique up to a bijection applied solely to k.

Let $f\colon n\to m$ be some injection, and consider the set $m\setminus \mathrm{im}(f)$ with $k=|m\setminus\mathrm{im}(f)|$. Notice that $m\setminus\mathrm{im}(f)$ is a finite subset of an ordered set m, so ordered again. This means that we can define a bijection $\psi\colon k\to m\setminus\mathrm{im}(f)$ by sending $\psi(1)$ to the least element of $m\setminus\mathrm{im}(f)$, $\psi(1)$, to the second-to-least element, and so on. Now define $\pi\colon n+k\to m$ by

$$\pi(a) = \begin{cases} f(a) & \text{if } a\le n\\ \psi(a-n) & \text{otherwise}\end{cases}$$

This is injective since f and ψ are injective and their images are disjoint by

$$\mathrm{im}(f)\cap\mathrm{im}(\psi) = \mathrm{im}(f)\cap(m\setminus\mathrm{im}(f)) = \emptyset,$$

and it is surjective since

$$\mathrm{im}(\pi) = \mathrm{im}(f)\cup\mathrm{im}(\psi) = \mathrm{im}(f)\cup(m\setminus\mathrm{im}(f)) = m$$

so bijective. But then since $\amalg_1 : n\to n+k$ is given by $\amalg_1(n)=n$, it follows by definition of π that $\pi\circ\amalg_1 = f$. Thus $\hat{F}$ is full.

DRAFT

To see that it is also faithful, suppose there exists some other $\pi'\colon n+k \to m$ such that $\pi' \circ \amalg_1 = f$ yet $\pi \neq \pi'$. Since $\pi' \circ \amalg_1 = f = \pi \circ \amalg_1$, it follows for all $a \leq n$ that

$$\pi(a) = \pi(\amalg_1(a)) = f(a) = \pi'(\amalg_1(a)) = \pi'(a)$$

so π and π' can only disagree on values of a where $n \leq a \leq n+k$, which must either way fall in the subset of the image $m \setminus \mathrm{im}(f)$. Consider now the bijection ${\pi'}^{-1} \circ \pi\colon n+k \to n+k$. By the argument above $({\pi'}^{-1} \circ \pi)(a) = a$ when $a \leq n$, and $({\pi'}^{-1} \circ \pi)(a) \geq n$ when $a \geq n$. Defining $\psi'(a) = ({\pi'}^{-1} \circ \pi)(a+n) - n$, we see that when $a \leq n$,

$$(\mathrm{id}_n \oplus \psi')(a) = \mathrm{id}_n(a) = a = ({\pi'}^{-1} \circ \pi)(a)$$

and when $a > n$ we have

$$(\mathrm{id}_n \oplus \psi')(a) = n + \psi'(a-n) = n + ({\pi'}^{-1} \circ \pi)((a-n)+n) - n = ({\pi'}^{-1} \circ \pi)(a)$$

so $\mathrm{id} \oplus \psi' = {\pi'}^{-1} \circ \pi$. But then unicity up to a bijection applied on k follows by

$$\pi' \circ (\mathrm{id}_n \oplus \psi') = \pi' \circ ({\pi'}^{-1} \circ \pi) = (\pi' \circ {\pi'}^{-1}) \circ \pi = \pi$$

which, in turn, means that $\hat{F}$ is faithful as well. □

A rig category is *coaffine* when any object A has a unique morphism from the additive unit O.

Theorem S19. *If* $\mathbf{C}$ *is an coaffine rig category, there exists a unique rig functor* $\mathbf{FinInj} \to \mathbf{C}$.

Proof. Since **FinPerm** is the initial rig category there exists a unique rig functor $\mathbf{FinPerm} \to \mathbf{C}$. Since $\mathbf{C}$ is coaffine, it follows by the universal property of the R-construction, that this unique functor factors uniquely as

$$\begin{array}{ccc} \mathbf{FinPerm} & \xrightarrow{\mathcal{E}} & R(\mathbf{FinPerm}) \\ & \overset{!}{\searrow} & \downarrow \hat{!} \\ & & \mathbf{C} \end{array}$$

This takes care of existence. For uniqueness, suppose there exists another rig functor $F\colon R(\mathbf{FinPerm}) \to \mathbf{C}$. We note that, by uniqueness of the rig functor $!\colon \mathbf{FinPerm} \to \mathbf{C}$, we must also have $! = F \circ \mathcal{E}$. By the Expansion-Raw Morphism factorisation, every morphism in $R(\mathbf{FinPerm})$ factors as a canonical injection $\amalg_1\colon n \to n+k$ followed by a bijection $\pi\colon n+k \to m$. By $! = F \circ \mathcal{E}$, F and $\hat{!}$ must agree on all bijections, so they can only every disagree on where they send the canonical injections $\amalg_i$. Since F and $\hat{!}$ are rig functors they must preserve the initial object, as it is the additive unit in both $R(\mathbf{FinPerm})$ and $\mathbf{C}$: thus, they must also preserve initial maps $0 \to A$, as these are unique in both $R(\mathbf{FinPerm})$ and $\mathbf{C}$. But then they must preserve $\amalg_1$ since $\amalg_1 = (\mathrm{id} \oplus !) \circ \rho_\oplus^{-1}$, where $\rho_\oplus\colon O \oplus A \to A$ is the coherence isomorphism, and F and $\hat{!}$ preserve all maps involved; so $F = \hat{!}$.

Since $R(\mathbf{FinPerm})$ and **FinInj** are rig isomorphic by Lemma S18, it follows that **FinInj** satisfies the same universal property. □

Another way of phrasing the theorem above is that **FinInj** is the free coaffine rig category. A diagram is called coaffine when it is made up of rig coherence isomorphisms and initial maps $0 \to A$.

Corollary S20. *An coaffine diagram that commutes in* **FinInj** *commutes in any other coaffine rig category as well.*

Classical control

Our last preparation before verifying the required axioms is to show that $\mathrm{Split}(\mathrm{LR}(\mathbf{C}))$ has classical control; more precisely, that $\oplus$ is a coproduct. The main idea is that in an coaffine rig category like $\mathrm{Split}(\mathrm{LR}(\mathbf{C}))$, we can record the outcome of a measurement in the object $I \oplus I$ as follows.

Definition S21. In a coaffine rig category, define a natural family of morphisms $\mu_{A,B}$:

$$\begin{array}{ccc} A \oplus B & \xrightarrow{\mu_{A,B}} & (A \oplus B) \otimes (I \oplus I) \\ \downarrow {\scriptstyle \rho_\otimes^{-1} \oplus \rho_\otimes^{-1}} & & \uparrow {\scriptstyle \delta_R^{-1}} \\ (A \otimes I) \oplus (B \otimes I) & \xrightarrow[(\mathrm{id} \otimes \amalg_1) \oplus (\mathrm{id} \otimes \amalg_2)]{} & (A \otimes (I \oplus I)) \oplus (B \otimes (I \oplus I)) \end{array}$$

DRAFT

Lemma S22. *The pentagon below commutes in any coaffine rig category.*

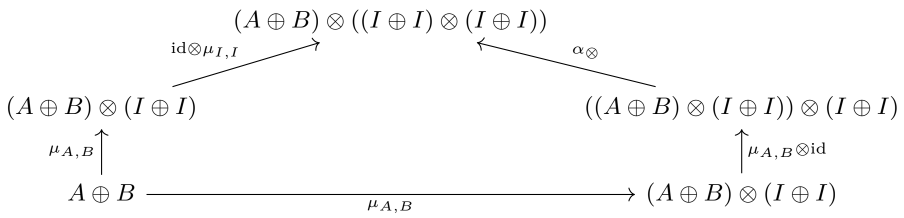

Proof. By commutativity of the diagram below.

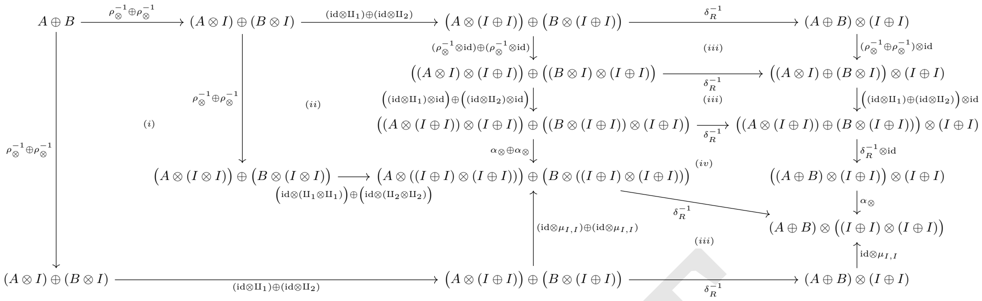

Here, (i) commutes by Corollary S20, (ii) by bifunctoriality of $\otimes$ and coherence, (iii) by naturality of δ_R^{-1}, and (iv) by coherence. □

Corollary S23. *When* $\mathbf{C}$ *is an coaffine rig category,* $\mu_{A,B}$ *is idempotent in* $L(\mathbf{C})$.

Proof. By the lemma above, $\mu_{I,I}$ mediates between $\mu_{A,B}$ and $\mu_{A,B} \circ \mu_{A,B}$. □

The intuition here is that $\mu_{A,B}$ measures whether the state is in the subspace A or B, yielding a single bit of information. That this bit can be mediated by $\mu_{I,I}$ means that we can either perform the same measurement again (getting the same result), or just copy the (classical) measurement outcome by measuring that.

Lemma S24. *The diagram below commutes in any coaffine rig category.*

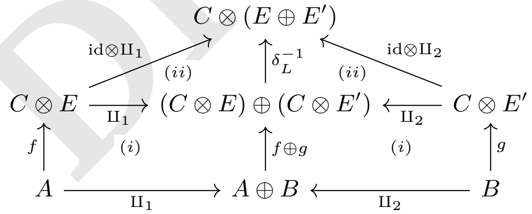

Proof. (i) commutes by naturality of injections, (ii) by Theorem S20. □

An object $A + B$ is a *coproduct* of A and B when it has injections $A \to A + B \leftarrow B$, and pairs of morphisms $A \to C \leftarrow B$ factor through a unique morphisms $A + B \to C$ via the injections. This codifies classical control categorically (1).

Proposition S25. $\mathrm{Split}(\mathrm{LR}(\mathbf{C}))$ *has coproducts.*

Proof. For objects (A, e) and (B, e') of $\mathrm{Split}(\mathrm{LR}(\mathbf{C}))$, we see that the diagrams

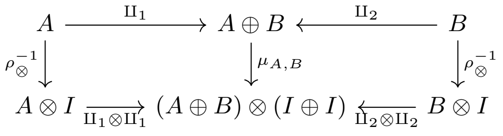

commute in $\mathrm{R}(\mathbf{C})$ by Corrolary S20, so $\mu_{A,B} \circ \amalg_1 = \amalg_1$ and $\mu_{A,B} \circ \amalg_2 = \amalg_2$ in $\mathrm{LR}(\mathbf{C})$; call their equivalence classes in $\mathrm{LR}(\mathbf{C})$ i_1 and i_2 respectively. Notice further that $(e \oplus e') \circ \mu_{A,B} = \mu_{A,B} \circ (e \oplus e')$ by naturality of $\mu_{A,B}$, so $(e \oplus e') \circ \mu_{A,B}$ is idempotent when e and e' are by

$$(e \oplus e') \circ \mu_{A,B} \circ (e \oplus e') \circ \mu_{A,B} = (e \oplus e') \circ (e \oplus e') \circ \mu_{A,B} \circ \mu_{A,B} = (e \oplus e') \circ \mu_{A,B}$$

Thus it follows by naturality of $\amalg_1$ and $\amalg_2$ that $(e \oplus e') \circ \amalg_1$ and $(e \oplus e') \circ \amalg_2$ are morphisms into $(A \oplus B, (e \oplus e') \circ \mu_{A,B})$ from (A, e) respectively (B, e'). Given morphisms $f : (A, e) \to (C, d)$ and $g : (B, e') \to (C, d)$ and choosing $[f, g]$ to be the equivalence class of $\delta_L^{-1} \circ (f \oplus g) \circ \mu_{A,B}$ in $\mathrm{LR}(\mathbf{C})$, we see that commutativity of the coproduct triangle

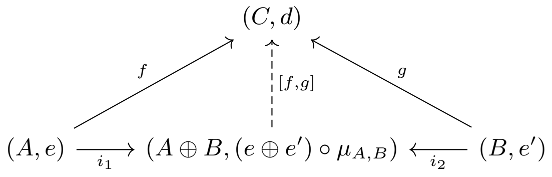

follows by Lemma S24 and $\amalg_1 = \amalg_1 \circ \mu_{A,B}$ and $\amalg_2 = \amalg_2 \circ \mu_{A,B}$ established above. Notice that $[f, g]$ respects the idempotents since

$$\begin{aligned}
& d \circ [f, g] \circ (e \oplus e') \circ \mu_{A,B} \\
&= d \circ \delta_L^{-1} \circ (f \oplus g) \circ \mu_{A,B} \circ (e \oplus e') \circ \mu_{A,B} \\
&= d \circ \delta_L^{-1} \circ (f \oplus g) \circ (e \oplus e') \circ \mu_{A,B} \circ \mu_{A,B} \\
&= \delta_L^{-1} \circ (d \oplus d) \circ (f \oplus g) \circ (e \oplus e') \circ \mu_{A,B} \circ \mu_{A,B} \\
&= \delta_L^{-1} \circ ((d \circ f \circ e) \oplus (d \circ g \circ e')) \circ \mu_{A,B} \circ \mu_{A,B} \\
&= \delta_L^{-1} \circ (f \oplus g) \circ \mu_{A,B} \circ \mu_{A,B} \\
&= \delta_L^{-1} \circ (f \oplus g) \circ \mu_{A,B} \\
&= [f, g]
\end{aligned}$$

in $\mathrm{LR}(\mathbf{C})$ by naturality of δ_L^{-1} and $\mu_{A,B}$, idempotence of $\mu_{A,B}$ and the fact that $d \circ f \circ e = f$ and $d \circ g \circ e'$ since they were assumed to be morphisms $(A, e) \to (C, d)$ and $(B, e') \to (C, d)$ in $\mathrm{Split}(\mathrm{LR}(\mathbf{C}))$, so this is indeed a morphism between the claimed objects in $\mathrm{Split}(\mathrm{LR}(\mathbf{C}))$. This takes care of existence.

To see that $[f, g]$ is also the unique such, suppose that h is some other morphism $(A \oplus B, (e \oplus e') \circ \mu_{A,B}) \to (C, d)$ making the coproduct triangle commute. By the fact that h is a morphism out of $(A \oplus B, (e \oplus e') \circ \mu_{A,B})$, we have that h respects both $\mu_{A,B}$ and $(e \oplus e')$ individually since

$$\begin{aligned}
h \circ \mu_{A,B} &= h \circ (e \oplus e') \circ \mu_{A,B} \circ \mu_{A,B} = h \circ (e \oplus e') \circ \mu_{A,B} = h \\
h \circ (e \oplus e') &= h \circ (e \oplus e') \circ \mu_{A,B} \circ (e \oplus e') = h \circ (e \oplus e') \circ (e \oplus e') \circ \mu_{A,B} \\
&= h \circ (e \oplus e') \circ \mu_{A,B} = h
\end{aligned}$$

in $\mathrm{LR}(\mathbf{C})$. But then

$$\begin{aligned}
h &= h \circ \mu_{A,B} \\
&= h \circ \mu_{A,B} \circ \mu_{A,B} \\
&= h \circ \delta_R^{-1} \circ ((\mathrm{id} \otimes \amalg_1) \oplus (\mathrm{id} \otimes \amalg_2)) \circ \rho_\otimes^{-1} \oplus \rho_\otimes^{-1} \circ \mu_{A,B} \\
&= h \circ \delta_L^{-1} \circ ((\amalg_1 \otimes \mathrm{id}) \oplus (\amalg_2 \otimes \mathrm{id})) \circ (\rho_\otimes^{-1} \oplus \rho_\otimes^{-1}) \circ \mu_{A,B} \\
&= \delta_L^{-1} \circ ((h \otimes \mathrm{id}) \oplus (h \otimes \mathrm{id})) \circ ((\amalg_1 \otimes \mathrm{id}) \oplus (\amalg_2 \otimes \mathrm{id})) \circ (\rho_\otimes^{-1} \oplus \rho_\otimes^{-1}) \circ \mu_{A,B} \\
&= \delta_L^{-1} \circ (((h \circ \amalg_1) \otimes \mathrm{id}) \oplus ((h \circ \amalg_2) \otimes \mathrm{id})) \circ (\rho_\otimes^{-1} \oplus \rho_\otimes^{-1}) \circ \mu_{A,B} \\
&= \delta_L^{-1} \circ ((f \otimes \mathrm{id}) \oplus (g \otimes \mathrm{id})) \circ (\rho_\otimes^{-1} \oplus \rho_\otimes^{-1}) \circ \mu_{A,B} \\
&= \delta_L^{-1} \circ (\alpha_\otimes \oplus \alpha_\otimes) \circ (\rho_\otimes^{-1} \oplus \rho_\otimes^{-1}) \circ (f \oplus g) \circ \mu_{A,B} \\
&= (\mathrm{id} \otimes \rho_\otimes^{-1}) \circ \delta_L^{-1} \circ (f \oplus g) \circ \mu_{A,B} \\
&= (\mathrm{id} \otimes \rho_\otimes^{-1}) \circ [f, g]
\end{aligned}$$

in $\mathrm{R}(\mathbf{C})$, so h and $[f, g]$ are in the same equivalence class in $\mathrm{LR}(\mathbf{C})$. □

Completeness

We are now ready to prove completeness of $\mathrm{Split}(\mathrm{LR}(\Pi_k))$ as in Theorem 5 in the main article. We will do so by verifying that it is a model for a signature satisfying the algebraic theory of quantum computation. For the axioms we refer to (7) and the proof of Theorem S26 and Theorem S28 below.

Proposition S26. *In $\mathrm{Split}(\mathrm{LR}(\mathbf{C}))$, with the exponential action of $I \oplus I$ on $\otimes$, the object I is a structure for the signature of the algebraic theory of quantum computation (7) (restricted to the unitaries of $\mathbf{C}$).*

DRAFT

Proof. Since Split($\mathbf{D}$) is symmetric monoidal when $\mathbf{D}$ is, it follows that Split(LR($\mathbf{C}$)) is symmetric monoidal as well (since $LR(\mathbf{C})$ is symmetric monoidal), and so admits an exponential action with $I \oplus I$ by Lemma S16. For the operations $\mathsf{new}\colon 0 \to 1$, $\mathsf{measure}\colon 1 \to (0,0)$, and $\mathsf{apply}_U\colon n \to n$ we associate the morphisms

$$\llbracket \mathsf{new} \rrbracket = \amalg_1 \colon I \to I \oplus I$$
$$\llbracket \mathsf{measure} \rrbracket = \mu_{I,I} \colon I \oplus I \to I + I$$
$$\llbracket \mathsf{apply}_U \rrbracket = F(U)\colon (I \oplus I)^{\otimes n} \to (I \oplus I)^{\otimes n}$$

where F is the symmetric monoidal functor $\mathbf{C} \to \mathrm{Split}(\mathrm{LR}(\mathbf{C}))$. These operations match the signature since $m \bullet I = (I \oplus I)^{\otimes m}$ in the exponential action of $I \oplus I$ (up to canonical coherence isomorphism). □

Definition S27. (7) A model of an algebraic theory with linear parameters is a structure for its signature such that for each axiom $x_1 : m_1, \ldots, x_k : m_k \mid a_1, \ldots, a_p \vdash t = u$ the interpretations $\llbracket t \rrbracket, \llbracket u \rrbracket : p \bullet X \to (m_1 \bullet X) + \cdots + (m_k \bullet X)$ are equal.

Theorem S28. *In* Split(LR($\mathbf{C}$)) *with the exponential action of* $I \oplus I$ *on* $\otimes$*, the object* I *forms a model of the algebraic theory of quantum computation (7).*

Proof. We verify the required axioms (A)-(L) (7). Write 2 for the object $1+1$, write δ^{l} and δ^{r} for the coherence isomorphisms $A \otimes (B \oplus C) \to (A \otimes B) \oplus (A \otimes C)$ and $(A \oplus B) \otimes C \to (A \otimes C) \oplus (B \otimes C)$, and write λ_A for the coherence isomorphism $I \otimes A \to A$.

The first group of axioms concerns coaffine diagrams, which are seen to hold in **FinInj** by direct computation, and therefore hold in Split(LR($\mathbf{C}$)) by Theorem S20:

(A) $\llbracket \mathsf{measure} \rrbracket \circ X = \mu_{1,1} \circ \sigma^+ = (\sigma^+ \times \sigma^+) \circ \mu_{1,1} = X \circ \llbracket \mathsf{measure} \rrbracket$;

(D) $\mathsf{measure} \circ \mathsf{new} = \mathsf{new}$;

(J) $(\delta^{\mathrm{r}} + \delta^{\mathrm{r}}) \circ \big((\mathsf{measure} \otimes \mathrm{id}) + (\mathsf{measure} \otimes \mathrm{id})\big) \circ \delta^{\mathrm{l}} \circ (\mathrm{id} \otimes \mathsf{measure})$
$= (\mathrm{id} + \sigma + \mathrm{id}) \circ (\delta^{\mathrm{l}} + \delta^{\mathrm{l}}) \circ \delta^{\mathrm{r}} \circ (\mathrm{id} \otimes \mathsf{measure}) \circ (\mathsf{measure} \otimes \mathrm{id})$;

(K) $(\llbracket \mathsf{new} \rrbracket \otimes \mathrm{id}_2) \circ (\mathrm{id}_2 \otimes \llbracket \mathsf{new} \rrbracket) = (\mathrm{id}_2 \otimes \llbracket \mathsf{new} \rrbracket) \circ (\llbracket \mathsf{new} \rrbracket \otimes \mathrm{id}_2)$;

(L) $(\mathrm{id}_2 \otimes \mathsf{measure}) \circ (\mathsf{new} \otimes \mathrm{id}_2) \circ \lambda_2^{-1} = (\mathsf{new} \otimes \mathrm{id}_2) \circ \lambda_2^{-1} \circ \mathsf{measure}$.

For example, the natural transformation $\mu_{A,B} \colon A + B \to (A+B) \times (1+1)$ is given by $A \ni a \mapsto (a, \amalg_1(*))$ and $B \ni b \mapsto (b, \amalg_2(*))$ in **FinInj**. Write

$$D(u,v) = (\delta^{\mathrm{r}})^{-1} \circ (u \oplus v) \circ \delta^{\mathrm{r}} \colon 2^{\otimes n+1} \to 2^{\otimes n+1}$$

for the map in LR($\mathbf{C}$) that models a computation on $n+1$ qubits, where the first qubit controls whether u or v is applied to the last n qubits. The following two axioms are proven via Theorem S20 as above using naturality.

(B) $D(u,v) \circ (\mathsf{measure} \otimes \mathrm{id}_{2^{\otimes n}}) = (\mathsf{measure} \otimes \mathrm{id}_{2^{\otimes n}}) \circ D(u,v)$;

(E) $D(u,v) \circ (\mathsf{new} \otimes \mathrm{id}_{2^{\otimes n}} \otimes \mathrm{id}_{2^{\otimes n}}) = \mathsf{new} \otimes u$.

The following group of axioms holds because the embedding $F\colon \mathbf{C} \to \mathrm{Split}(\mathrm{LR}(\mathbf{C}))$ is by construction a symmetric monoidal functor:

(F) $F(\sigma^{\otimes}_{2^{\otimes m}, 2^{\otimes n}}) = \sigma^{\otimes}_{2^{\otimes m}, 2^{\otimes n}}$;

(G) $F(\mathrm{id}_{2^{\otimes n}}) = \mathrm{id}_{2^{\otimes n}}$;

(H) $F(v \circ u) = F(v) \circ F(u)$;

(I) $F(u \otimes v) = F(u) \otimes F(v)$;

The final axiom follows from the nature of the construction of Split(LR($\mathbf{C}$)):

(C) It follows from the fact that I is terminal in Split(LR($\mathbf{C}$)) that $d \circ u = d$ for any $u\colon 2^{\otimes n} \to 2^{\otimes n}$ and $d\colon 2^{\otimes n} \to I$ in $\mathbf{C}$, so in particular for the discarding map $d\colon 2^{\otimes n} \to 1$ in Split(LR($\mathbf{C}$)) lifted from $\lambda^{-1}\colon 2^{\otimes n} \to I \otimes 2^{\otimes n}$ in $\mathbf{C}$. □

Let **QChannel** denote the category of finite-dimensional C^*-algebras and quantum channels (completely positive trace-preserving linear maps). The following corollary to Theorem S28 establishes the first part of Theorem 5 in the main article.

Corollary S29. *The induced functor* $\llbracket - \rrbracket \colon \mathrm{Split}(\mathrm{LR}(\Pi_k)) \to$ **QChannel** *is faithful.*

Proof. It was established in (7) that every equality of quantum channels can be established using the discrete set of axioms shown to hold in Split(LR(Π_k)) by Theorem S28. □

Finally, we conclude that Split(LR(Π_k)) is complete for unitaries, establishing the last part of Theorem 5 in the main article.

DRAFT

Theorem S30. *Parallel morphisms f and g in Π_k are equal when included into* $\mathrm{Split}(\mathrm{LR}(\Pi_k))$ *if and only if $f \approx_k s \cdot g$ for some scalar s.*

Proof. Suppose $f \approx_k s \cdot g$ in Π_k, i.e., there exist f' and g' such that $f \oplus f' = (s \cdot g) \oplus g'$. Note that $f \oplus f'$ and $(s \cdot g) \oplus g'$ are precisely interpretations of the circuits

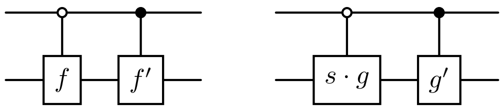

But then $f = g$ in $\mathrm{Split}(\mathrm{LR}(\Pi_k))$ follows by

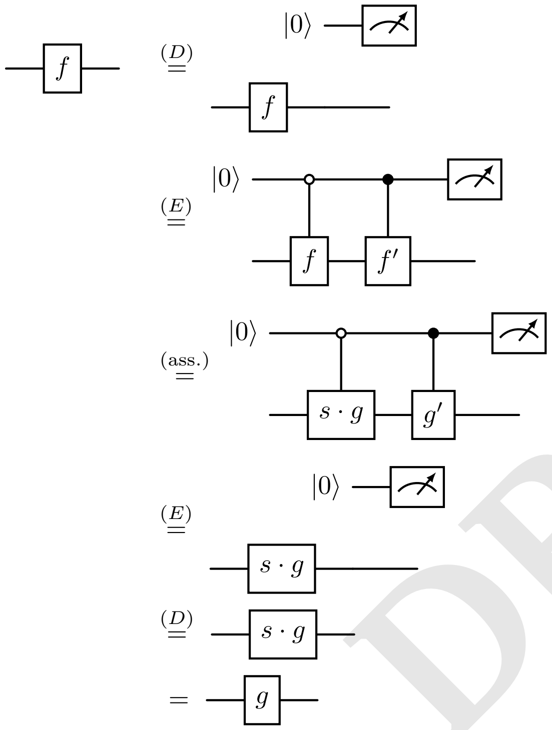

□

where the last step follows as all scalars are identified in $\mathrm{Split}(\mathrm{LR}(\Pi_k))$ (since I is terminal), so $s \cdot g = \mathrm{id} \cdot g = g$.

In the other direction, when f and g are unitaries in Π_k such that $f = g$ when included in $\mathrm{Split}(\mathrm{LR}(\Pi_k))$, it follows by functoriality of $[\![-]\!]\colon \mathrm{Split}(\mathrm{LR}(\Pi_k)) \to$ **QChannel** that $[\![f]\!] = [\![g]\!]$, and since f and g were included from Π_k these quantum channels in fact just adjoining by the underlying unitaries of f and g. Since adjoining by the underlying unitaries of f and g give equal quantum channels, it follows by unitary freedom (14, Theorem 2.6) of their Choi-states that their underlying unitaries are equal up to a global phase. Applying Theorem S15 then establishes $f \approx_k s \cdot g$ in Π_k.

Asymptotic scaling with k

We study how the Π_k term size needed to approximate a target unitary depends on the precision level k. Given an overall approximation error ε in operator norm, how does the minimal Π_k term size scale with k for different classes of unitaries? Two representative cases illustrate the range of behaviours: generic unitaries and the quantum Fourier transform.

Generic unitaries. Consider circuits made of single-qubit rotations and CNOT gates. A rotation $R_a(\phi)$ can be exactly represented by a term of Π_k if and only if the entries of $R_a(\phi)$ lie in $\mathbb{D}[\zeta_k]$ (15); all other rotations must be approximated. For such generic rotations, any ε-approximation requires $O(\log(1/\varepsilon))$ primitive terms and approximations matching this lower bound can be constructed efficiently (16). If a constant fraction of the rotations in a circuit are generic, the overall size of the corresponding Π_k term therefore scales as $O(\log(1/\varepsilon))$ for any fixed k, with larger k potentially improving only the hidden constant factor. Thus, no finite k can remove the logarithmic dependence on ε, only improve its constant.

DRAFT

The quantum Fourier transform. The n-qubit quantum Fourier transform (QFT_n) contains n Hadamard gates and $\binom{n}{2}$ controlled-phase gates. The angles appearing in the phase gates are of the form $2\pi/2^d$, with d ranging from 2 to n. At precision level k, all rotations with $d \leq k$ are native to Π_k and therefore require no approximation. Hence, when $n \leq k$, no approximations are needed at all, and $O(n^2)$ Π_k-terms suffice to represent QFT_n exactly. When $n > k$,

$$\binom{(n-k)+1}{2}$$

controlled-phase gates cannot be exactly represented in Π_k and must therefore be approximated. For example, a single gate requires approximation when $k = n-1$, three gates require approximations when $k = n-2$, and so on. Overall, assuming that the total error budget ε is split evenly among all gates requiring approximation, standard methods (16) will therefore produce Π_k terms of size $O((n^2-(n-k)^2)+(n-k)^2\log_2((n-k)^2/\varepsilon)))$. Hence, in the case of the quantum Fourier transform, increasing k can remove the dependence on ε altogether: As k increases, the residual cost drops until $k = n$, after which all rotations are native and the size plateaus at $O(n^2)$.

1. C. Heunen and J. Vicary. *Categories for Quantum Theory*. Oxford University Press, Oxford, 2019.
2. J. P. May. *E_∞ Ring spaces and E_∞ ring spectra*. Springer, Heidelberg, 1977.
3. M. L. Laplaza. Coherence for distributivity. In *Coherence in Categories*, pages 29–65, Heidelberg, 1972. Springer.
4. S. Mac Lane. Categorical algebra. *Bulletin of the American Mathematical Society*, 71:40–106, 1965.
5. E. G. Cate and D. W. Twigg. Analysis of in-situ transposition. *ACM Transactions on Mathematical Software*, 3:104–110, 1977.
6. J. Carette, C. Heunen, R. Kaarsgaard, and A. Sabry. With a few square roots, quantum computing is as easy as Π. In *Proceedings ACM on Programming Languages*, volume 8, page 564, 2024.
7. S. Staton. Algebraic effects, linearity, and quantum programming languages. In *Proceedings ACM on Programming Languages*, pages 395–406, 2015.
8. C. Heunen and R. Kaarsgaard. Quantum information effects. In *Proceedings ACM on Programming Languages*, volume 6, pages 1–27, 2022.
9. P. Andres-Martinez, C. Heunen, and R. Kaarsgaard. Universal properties of partial quantum maps. In *Quantum Physics and Logic*, volume 394 of *Electronic Proceedings in Theoretical Computer Science*, pages 192–207, 2023.
10. W. H. Zurek. Decoherence and the transition from quantum to classical. *Physics Today*, 10:36–44, 1991.
11. P. Selinger. Idempotents in dagger categories. In *Quantum Physics and Logic*, volume 210 of *Electronic Notes in Theoretical Computer Science*, pages 107–122, Amsterdam, 2008. Elsevier.
12. G. Janelidze and G. M. Kelly. A note on actions of a monoidal category. *Theory and Applications of Categories*, 9:61–91, 2001.
13. C. Hermida and R. D. Tennent. Monoidal indeterminates and categories of possible worlds. *Theoretical Computer Science*, 430:3–22, 2012.
14. M. A. Nielsen and I. L. Chuang. *Quantum computation and quantum information*. Cambridge University Press, Cambridge, 2010.
15. Vadym Kliuchnikov, Dmitri Maslov, and Michele Mosca. Asymptotically optimal approximation of single qubit unitaries by clifford and T circuits using a constant number of ancillary qubits. *Phys. Rev. Lett.*, 110:190502, May 2013. . URL https://link.aps.org/doi/10.1103/PhysRevLett.110.190502.
16. N. J. Ross and P. Selinger. Optimal ancilla-free Clifford+T approximation of z-rotations. *Quantum Information and Computation*, 16:901–953, 2016.

DRAFT
